# Genome-wide identification and characterization of cytochrome P450 monooxygenase genes in the ciliate *Tetrahymena thermophila*

**DOI:** 10.1186/1471-2164-10-208

**Published:** 2009-05-01

**Authors:** Chengjie Fu, Jie Xiong, Wei Miao

**Affiliations:** 1 Key Laboratory of Aquatic Biodiversity and Conservation, Institute of Hydrobiology, Chinese Academy of Sciences, Wuhan, 430072, PR China; 2 State Key Laboratory of Freshwater Ecology and Biotechnology, Institute of Hydrobiology, Chinese Academy of Sciences, Wuhan, 430072, PR China; 3 Graduate School of Chinese Academy of Sciences, Beijing, 100049, PR China

## Abstract

**Background:**

Cytochrome P450 monooxygenases play key roles in the metabolism of a wide variety of substrates and they are closely associated with endocellular physiological processes or detoxification metabolism under environmental exposure. To date, however, none has been systematically characterized in the phylum Ciliophora. *T. thermophila *possess many advantages as a eukaryotic model organism and it exhibits rapid and sensitive responses to xenobiotics, making it an ideal model system to study the evolutionary and functional diversity of the P450 monooxygenase gene family.

**Results:**

A total of 44 putative functional cytochrome P450 genes were identified and could be classified into 13 families and 21 sub-families according to standard nomenclature. The characteristics of both the conserved intron-exon organization and scaffold localization of tandem repeats within each P450 family clade suggested that the enlargement of *T. thermophila *P450 families probably resulted from recent separate small duplication events. Gene expression patterns of all *T. thermophila *P450s during three important cell physiological stages (vegetative growth, starvation and conjugation) were analyzed based on EST and microarray data, and three main categories of expression patterns were postulated. Evolutionary analysis including codon usage preference, site-specific selection and gene-expression evolution patterns were investigated and the results indicated remarkable divergences among the *T. thermophila *P450 genes.

**Conclusion:**

The characterization, expression and evolutionary analysis of *T. thermophila *P450 monooxygenase genes in the current study provides useful information for understanding the characteristics and diversities of the P450 genes in the Ciliophora, and provides the baseline for functional analyses of individual P450 isoforms in this model ciliate species.

## Background

The cytochrome P450 monooxygenases (P450s) constitute a conserved gene superfamily of heme-thiolate proteins ubiquitously distributed in different life forms, including prokaryotes (archaea, bacteria), unicellular eukaryotes (protists, fungi) and multicellular eukaryotes (plants and animals) [1]. They play key roles in the metabolism of a wide variety of substrates, including endogenous chemicals such as steroids and other important small molecules, and also xenobiotic compounds including drugs, pesticides and environmental contaminants [2]. Substrate and functional diversity are characteristic features of P450 enzymes and are considered to be the consequence of evolutionary adaptation driven by different metabolic or environmental demands in different organisms. Evolution and expansion of major P450 branches has been suggested to be linked with the historical occurrence of important evolutionary events. One particular example is the divergence of P450s of the common plant-animal ancestor either to synthesize biochemicals/metabolites (in plants) or to detoxify xenobiotics (in animals), followed by P450 gene expansions, especially in the plants [3]. These may well reflect different survival strategies adopted between the two kingdoms, i.e. plants evolved sessile systems with P450 enzymes with more diverse and essential roles, while animals developed higher order sensory and locomotor systems, and comparatively fewer P450s [4].

Although over 9,000 P450s have been named and about 1,000 P450 genes have been characterized to date, none has been systematically characterized in the phylum of Ciliophora which, together with dinoflagellates and the exclusively parasitic apicomplexa, constitute the three major evolutionary lineages that make up the alveolates [5]. The unicellular ciliate *Tetrahymena thermophila *is a free-living protozoan widely distributed in freshwater and estuarine environments, elaborating typical eukaryotic components (e.g., microtubules, membrane systems) into a highly organized cell whose structural and functional complexity is comparable to, or exceeds that, of human and other metazoan cells [6]. The physiology, biochemistry and molecular biology of *Tetrahymena *are well characterized [7], and it is an excellent model organism for toxicological and ecotoxicological studies in aquatic toxicity test systems [8,9]. Results from the EST project [10] and the macronuclear genome sequencing project [11] have shown that, although single-celled, *Tetrahymena *possesses core processes conserved across a wide diversity of eukaryotes (including humans) that are not found in other unicellular model species such as the yeasts *Saccharomyces cerevisiae *and *Schizosaccharomyces pombe*. It also contains a large number of gene families that are involved in processes associated with sensing and responding to environmental cues. In the case of the cytochrome P450 gene family, *S. cerevisiae *and *S. pombe *have only three and two P450s, respectively [4], while in *T. thermophila *the number is more than 40 (this study), which is close to the typical number (50–80) in a vertebrate genome [12].

In humans and other mammals, extensive studies have focused on aspects of P450 gene structure and biochemical properties. Important biological functions of P450s and the associated high degree of complexity in the gene polymorphism and expression patterns have been demonstrated [13]. As genomic databases became available and functional genomics techniques such as DNA-microarrays have been applied, investigations on P450 isoforms have also been extended to other organisms (birds, crustaceans, insects, fungi, plants) (see [14] for a recent review). These developments have, in turn, led to the emergence of a new field, ecotoxicogenomics [15], which aims to develop effective tools for identification of possible toxic environmental pollutants by characterizing their effects on terrestrial and aquatic model organisms, such as the soil-dwelling nematode *Caenorhabditis elegans *[16], the aquatic crustacean *Daphnia magna *[17], and the small fish fathead minnow (*Pimephales promelas*) [18].

We previously identified a series of differentially expressed ESTs of *T. thermophila *that respond sensitively to treatment with the organochlorine insecticide DDT [19]. One EST (GenBank accession No. CF653700) was identified to be a P450 gene by homology searches, and its expression levels under different concentrations of DDT treatment were further assessed. Recently, the first genome-wide microarray platform containing the predicted coding sequences (putative genes) has been established and validated in *T. thermophila *and was used to study gene expression during three major stages of the organism's life cycle: vegetative growth, nutrient starvation and conjugation [20]. Substantial progress has also been made in closure and reannotation of the MAC genome sequence of this eukaryotic model organism [21]. All these provided us the opportunity to investigate both the functional and evolutionary characteristics of the cytochrome P450 genes in *T. thermophila *at the genomic level.

In this study, the putative *T. thermophila *P450 genes that were previous identified both by the International Nomenclature Committee (Nelson's P450 Homepage ) and by the TIGR genome annotation team were further checked by EST data and through cDNA sequence cloning experiments for improvement of annotation. The expression patterns of the *T. thermophila *P450 genes during three important cell physiological/developmental conditions (growth, starvation and conjugation) were analyzed based on EST and microarray data. These results are discussed in the context of understanding the characteristics of the *T. thermophila *P450 monooxygenase isoforms and their functional and evolutionary diversity.

## Results and discussion

### *T. thermophila *putative P450 gene sequences

The putative cytochrome P450 gene sequences in *T. thermophila *were initially identified in 2004 by Dr. Nelson based on an early TIGR release of its macronuclear genome assemblies, and 47 P450-like genes (and fragments) were posted on his P450 website . When the draft whole genome of *T. thermophila *was sequenced and reported in 2006, 41 P450 genes were predicted by the TIGR genome annotation team [11]. In this study, we retrieved the putative *T. thermophila *P450 genes from above two locations and further searched against the TGD (Tetrahymena Genome Database) utilizing the updated annotation (Aug. 2007). The obtained P450-like gene sequences were thereafter compared. Then, we used the data from two EST database resources (NCBI and TBestDB) and our own RT-PCR investigations to verify the accuracy of the predictions.

A total of 44 putative cytochrome P450 gene sequences with full-length ORFs were identified in *T. thermophila *and were assigned names as suggested by the "P450 nomenclature committee" (Table 1). Our results showed that the gene predictions made by Nelson are more accurate than those presented by the TIGR annotation. The TIGR predictions have some errors in identifying the start codons, especially the signal peptide, and intron-exon boundaries of several P450s are inaccurate. In one case, it merged 3 adjacent P450 genes (*CYP5013A1*, *CYP5013C1 *and *CYP5013B1*) into one "monster" gene (3,150 amino acids, 9.5 kb and 27 exons), which also includes two intergenic regions plus 1.3 kb upstream of *CYP5013A1 *(Additional file 1). For many other P450 genes, the TIGR annotations are systematically short, presumably due to its failure to recognize the signal peptide at the 5' end. This problem may arise if the hydrophobic region is coded by AT-rich codons, which are not easily distinguished statistically from the AT-rich intergenic regions (E. Orias, Personal Communication). For all the 44 P450 genes, only three sequences (*CYP5008A1*, *CYP5011A1 *and *CYP5012A1*) were modified slightly in our annotation compared with the predictions made by Dr. Nelson.

**Table 1:** The 44 T. thermophila putative P450 genes.

Gene ID	GenBank accession No.	Scaffold GenBank accession No.	Loc	EST accession No.
CYP5001A1	TTHERM_00408880	CH445583	/	
CYP5002A1	TTHERM_00516350	CH445658	S	DY683840/CX578941/CX580685
CYP5003A1	TTHERM_00112850	CH445735	S	
CYP5004A1	TTHERM_00191380	CH445644	S	
CYP5005A1	TTHERM_00198200	CH445786	S	
CYP5005A2	TTHERM_00198210	CH445786	S	
CYP5005A3	TTHERM_00198220	CH445786	S	
CYP5005A4	TTHERM_00198230	CH445786	S	
CYP5005A6	TTHERM_00198320	CH445786	S	
CYP5005A7	TTHERM_00198340	CH445786	S	EC269404
CYP5005A8	TTHERM_00200550	CH445786	S	
CYP5005A9	TTHERM_00201580	CH445786	S	
CYP5005A10	TTHERM_00201630	CH445786	S	CX583778/CX575364
CYP5005A14	TTHERM_00227020	CH445668	S	
CYP5005A15	TTHERM_00898320	CH445574	S	
CYP5005A16	TTHERM_00101170	CH445709	S	
CYP5005A17	TTHERM_01398470	CH670446	S	CX579342
CYP5005A18	TTHERM_01122770	CH445602	S	EC269907/DY677658/DY677657/EC269908
CYP5005A19	TTHERM_01122780	CH445602	S	DY679355/DY679356/CX580561/EC269178/EC269178/CX583603/CX583579
CYP5005A20	TTHERM_01369770	CH445637	S	
CYP5006A1	TTHERM_00185610	CH445770	S	DY677831/DY677832
CYP5007A1	TTHERM_00620930	CH445621	S	
CYP5007B1	TTHERM_00283410	CH445618	S	
CYP5007C1	TTHERM_00283420	CH445618	S	EC274613/EC274614
CYP5008A1	TTHERM_00101290	CH445709	S	CN592969/DY679809/CN593111/BM400871/CF653700
CYP5008A2	TTHERM_01280630	CH445497	S	TTL00012665*
CYP5009A1	TTHERM_00444460	CH445552	S	
CYP5010A1	TTHERM_00723150	CH670361	S	
CYP5010A2	TTHERM_01250020	CH445573	S	
CYP5010A3	TTHERM_01698320	CH445507	S	DY683602
CYP5010A4	TTHERM_00754730	CH445616	S	
CYP5010A5	TTHERM_00754700	CH445616	S	DY683602/CX586098
CYP5010B1	TTHERM_00129890	CH445650	S	DY683352/CX591511/CX571835/BM399027
CYP5010C1	TTHERM_01415170	CH670449	S	
CYP5010C2	TTHERM_00449550	CH445687	S	
CYP5011A1	TTHERM_00527100	CH445398	S	CX588690
CYP5012A1	TTHERM_00137750	CH445601	S	TTL00011142*
CYP5012A2	TTHERM_01250020	CH445573	S	
CYP5013A1	TTHERM_00437540	CH445623	S	DY677981/EC271015/BM400694/EC271842/EC272511/EC270080/EC270079/CX585070/CX584716/CX579033/DY677982/CX577930/DY680538/CX574127/EC272512/BM399152/CX577367/CX579948/DY678167/CX572624
CYP5013B1	TTHERM_00437540	CH445623	S	
CYP5013C1	TTHERM_00437540	CH445623	S	
CYP5013C2	TTHERM_00241770	CH445533	S	
CYP5013D1	TTHERM_00395750	CH445712	S	BM399816/BM396441/BM399815
CYP5013E1	TTHERM_00313500	CH670346	S	EC269139/EC270428

Gene ID, GenBank accession numbers, Scaffold GenBank accession numbers, EST accession numbers and predictions of sub-cellular localizations were listed. S: secretory pathway. *: Found only in TBestDB.

There were four P450 pseudogenes (fragments) in Nelson's predictions (*CYP5005A5P*, *CYP5005A7P*, *CYP5005A12P *and *CYP5005A13P*, "*P*" stands for "pseudo"). It was revealed that one (*CYP5005A5P*) was a probable pseudogene due to the presence of in-frame stop codons, and two partial fragments (*CYP5005A12P *and *CYP5005A13P*) with typical P450 sequence features appeared to be missing the N-terminus. These sequences were not included in the following analysis of the *T. thermophila *putative P450 genes. However, when checking the cDNA sequences of putative *T. thermophila *P450 genes, we identified one transcription product that is most identical to the predicted pseudogene "*CYP5005A7P*" which has an in-frame TGA codon (the only stop codon in *T. thermophila*) within its ORF. Compared with the "*CYP5005A7P*" sequence, the EST of the cDNA transcript has several site mutations including one nucleotide transition (A to G) within the in-frame stop codon found in the genomic sequence, and thus can be read through. However, a Blast search of the *T. thermophila *whole genome database failed to detect any sequence that completely matched this cDNA sequence. Thus we designed a pair of primers (5005A7_Genome_Fw and 5005A7_Genome_Re) located in either the 5' or 3' flanking region of the "*CYP5005A7P*" sequence, and obtained a 2.1kb PCR amplification product that was sequenced. Due to the possibility that different P450 isoforms may exist in different *T. thermophila *strains, we checked the PCR products both from the genomic DNA of strain SB210 (the strain that used for the *T. thermophila *macronuclear sequencing project) and CU428. The results showed that the PCR products from the two strains were 100% identical and were also consistent with the sequence of the unexpected cDNA transcript. We thus assigned the name *CYP5005A7 *to this newly observed P450 isoform. Further, Blast searches in the TBestDB and NCBI EST database either found one sequence in each that perfectly matched the *CYP5005A7 *sequence (TBestDB accession No. TTL00012823, cDNA library from a *T. thermophila *CU strain; GenBank accession No. EC269404, cDNA library from the *T. thermophila *strain SB210, respectively). Besides, the microarray data demonstrated that the corresponding cDNA transcript was constantly expressed during the different cellular conditions. All the above observations serve as evidence that *CYP5005A7 *is a functional P450 gene in both the *T. thermophila *CU and SB210 strains. The erroneous prediction of the pseudogene "*CYP5005A7P*" by TIGR was probably due to false assembly of genome sequence contigs, caused by the high similarity between the *CYP5005A5P *and *CYP5005A7 *sequences (Additional file 2).

### Structural features and intron-exon organization

**Figure 1:**
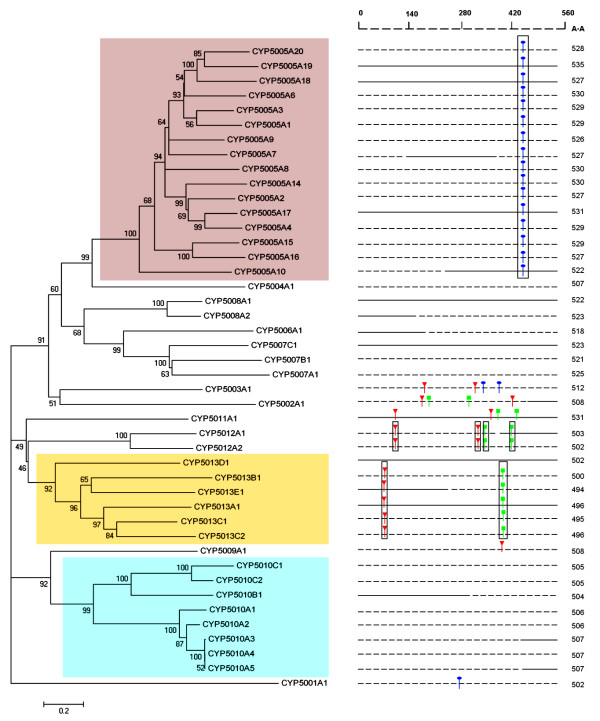
**Phylogenetic tree of *T. thermophila *P450 genes with intron location analysis**. Left: The NJ tree with bootstrap values. Right: Predicted P450 gene sequences were drawn as dotted lines, gene sequences with available ESTs were drawn as straight lines. A phase zero splice site lies between two codons, while a phase I splice site lies one base inside a codon in the 3' direction, and a phase II intron is two bases inside a codon in the 3' direction. Intron organization: Green, phase zero; Red, phase I; Blue, phase II. Consensus Introns that share the same aligned position are enclosed in a black square. The three largest families are enclosed in the colored squares.

P450 genes are classified and annotated on the basis of amino acid sequence identity, phylogenetic homology and gene organization, and a four-digit naming system has been set up to meet the need of the increasing number of newly discovered P450 sequences. All the *T. thermophila *P450 sequences are distributed into 13 families and 21 sub-families according to the nomenclature criterion of sequence similarity (>40% as a family and >55% as a sub-family) [22], indicating P450s exist as a large gene family in this organism. The phylogenetic analysis of *T. thermophila *P450 genes obtained using either a neighbor-joining tree or a maximum-likelihood tree gave similar topological nodes, and bootstrap testing showed good reliability of the phylogenetic tree (Figure 1, Additional file 3).

The largest expanded P450 family in *T. thermophila *is the CYP5005 family that contains 16 likely functional P450 genes (purple in Figure 1) and the two predicted pseudogenes (not indicated). The CYP5010 family (blue in Figure 1) and the CYP5013 family (orange in Figure 1) were "medium"-expanded P450 families in *T. thermophila *that possessed 8 and 6 genes, respectively. Moreover, 10 CYP5005 family members are located on the same scaffold (CH445786), which contains a gene cluster in the form of tandem repeats with four genes (*CYP5005A1*, *CYP5005A2*, *CYP5005A3 *and *CYP5005A4*) and one pseudogene (*CYP5005A5P*) in the same orientation. Three CYP5013 family members (*CYP5013A1*, *CYP5013B1 *and *CYP5013C1*) form a tandem triplicate repeat on the scaffold CH445623. Besides these, there are three other P450 gene pairs (*CYP5005A18 *and *CYP5005A19*, *CYP5007B1 *and *CYP5007C1*, *CYP5012A1 *and *CYP5012A2*) organized in tandem duplication. Such a feature is consistent with the gene expansion strategy used by some other lower eukaryotic genomes, particularly those who have evolved to meet an extensive demand for generation of a broad range of metabolites in the secondary metabolism when interacting with the environmental niches. For example, it was revealed that the majority of the multigene P450 families in the model white rot fungus *Phanerochaete chrysosporium *appear to have expanded locally in its genome as a result of extensive gene duplications and rearrangements, indicating a strong need for functional divergence in response to environmental stimuli [23].

The intron-exon organization of P450 genes exhibits a diversity of gene structure with indicating that multiple gains and losses of introns have occurred during the evolution of P450 genes in diverse species, with little conservation of intron positions among divergent P450 families [24,25]. Intron positions were mapped and characterized for all 44 putative *T. thermophila CYP *genes (Figure 1). Of the 48 introns that were identified in the P450 gene sequences, 13 were phase zero (27.1%), 16 were phase I (33.3%) and 19 were phase II (39.6%). Meanwhile, based on the definition of the UIP (introns that occupy a unique position in the alignment) [26], 27.1% (13) of the total introns were unique and the remaining 35 introns were present in 7 consensus locations (introns shared by all members within a family at the same aligned position) among three different family clades. Sixteen P450 genes lack introns entirely. Five genes (*CYP5002A1*, *CYP5003A1*, *CYP5011A1*, *CYP5012A1 *and *CYP5012A2*) have the maximum number of four introns, respectively. From Figure 1, it can be observed that there is a good correlation between the conservation of intron position and phylogenetic relationships of *T. thermophila *P450 subfamily members. The evolution of introns in alveolates was recently studied by Nguyen et al. [27], who concluded that the rates of intron gain and loss were more or less constant in the last ~800 Myr after *Tetrahymena *branched off. Therefore, it can be inferred that the enlargement of several P450 families in *T. thermophila *resulted from recent separate small duplication events, which is also apparent within many other *Tetrahymena *protein families containing paralogous genes [11].

Our analyses of the EST data to determine the intron boundaries also revealed that the *CYP5013A1 *gene might exhibit alternative intron splicing. Alternative splicing was suggested to be an uncommon phenomenon in *T. thermophila*, at least under the several growth conditions that have examined [21]. *CYP5013A1 *had the greatest number of ESTs of all *T. thermophila *P450 isoforms. Among the 26 retrievable EST sequences, 25 represented the correct transcriptional product of *CYP5013A1*, while one (GenBank accession No. FF565796) retained the first intron. The different transcripts were further investigated by RT-PCR. An intron-containing transcript was observed in PCR products both from the cDNA templates of starvation and conjugation stages, but was barely detectable from vegetative growing cells (data not shown). The retained intron in *CYP5013A1 *is 53 bp in length and belongs to phase I type. Thus, the putative intron-containing transcript should contain a frame-shift mutation. RNA transcripts carrying such premature stop codons can be prevented by the NMD pathway [28]. Recently, this pathway was found to play an essential role in another ciliate, *P. tetraurelia*, by knocking down the two homologous genes encoding UPF1, a protein which is crucial in NMD, thus indicating a universal translational control of intron splicing in eukaryotes based on NMD surveillance [29]. A blast search of the *T. thermophila *genome using *P. tetraurelia *UPF1 genes as queries revealed the existence of one homolog with 70% identity (GenBank accession No. TTHERM_00726300). Thus, intron retention in the *CYP5013A1 *transcript might be caused by inefficient NMD activity during specific physiological/developmental stages. However, it would lead to a mature "non-sense" transcript and could only serve to down-regulate expression of the *CYP5013A1 *gene. Whether such rarity of alternative splicing in this species may simply be tolerated or directed by other mechanisms, is currently unknown.

**Figure 2:**
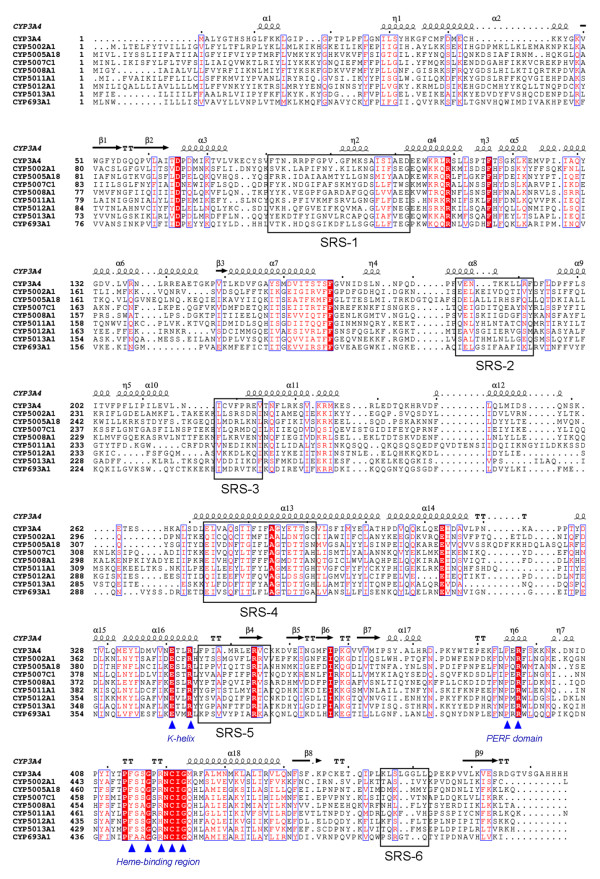
**Multiple sequence alignment and secondary structure elements assignment**. The alignment of 7 representative *T. thermophila *P450 genes and one predicted *P. tetraurelia *P450 gene (*CYP693A1*, ParameciumDB accession No. GSPATP00036495001) with the mamalian CYP3A4 protein sequence. Substrate recognition sites (SRSs) 1–6 were manually determined. The heme-binding region (FXXGXRXCXG), PERF domain (PXRX) and K-helix (EXXR) were indicated with blue triangles respectively. The alpha helices are marked as alpha or eta based on the automatic assignment according to the template of CYP3A4 protein structure in the program ESPript.

All known P450s appear to take on a similar folded structure, yet frequently show less than 30% sequence identity and have very different substrates [30]. In *T. thermophila*, the average length of the P450 amino acid sequences in the alignment is around 500 amino acid residues (Figure 1), and the genes share different degrees of amino acid sequence conservation between different families. To better understand the structure and functional relationship of P450 enzymes, mammalian P450 CYP3A4, seven *T. thermophila *P450 sequences (*CYP5002A1*, *CYP5005A18*, *CYP50077C1*, *CYP5008A1*, *CYP5011A1*, *CYP5012A1 *and *CYP5013A1*) and one *Paramecium tetraurelia *P450 sequence (*CYP693A1*, ParameciumDB accession No. GSPATP00036495001) were selected and aligned (Figure 2). Secondary elements were then assigned using CYP3A4 as the template for secondary structure, based on its known crystal structures and six putative SRSs were identified in the aligned sequences according to Gotoh's predicted models [31]. SRSs participate in the contact with ligands and many of them locate at regions with variable structural elements. All the *T. thermophila *P450 sequences contain the typical conserved P450 domains, including the heme-binding region (FXXGXRXCXG) near the C-terminal, the PERF domain (PXRX) and the K-helix (EXXR). Multiple sequence alignment also revealed that some residues are identical across *T. thermophila *P450s; most of these are distributed in the five regions that constitute the conserved P450 structural core, including: the C helix (α4), the C-terminal part of the E helix (α7), the I helix (α13), the J helix (α14), and the heme-binding loop between helix K (α16) and L (α18) (Figure 2, Additional file 4).

### *T. thermophila *P450 member composition

Prediction of sub-cellular localization of the CYP proteins showed that no predicted mitochondrial localized P450 was found in *T. thermophila *(Table 1), and all of the genes except *CYP5001A1 *encode a putative typical microsomal signal peptide of about 20 hydrophobic residues that likely serves as a membrane anchor in the endoplasmic reticulum. The secretory pathway of eukaryotic cells consists of a series of discrete, membrane-bound compartments, including the ER, Golgi, and vacuoles [32], all of which are present in *Tetrahymena *[7]. For *CYP5001A1*, the predicted signal sequence was ambiguous and also lacked the positively charged residues in this N-terminus critical for endocellular transportation targeting. So far, mitochondrial P450s have been described only in the animal kingdom [4,25]. As with *T. thermophila*, none was found in fungi and plants. These observations support the suggestion that mitochondrial P450s did not originate from the ancestral mitochondrial endosymbiont, but evolved later, possibly by mistargeting of microsomal P450s [33].

CYP51 is the only P450 family that has orthologs in multiple phyla of the animal, plant, fungal and bacterial kingdoms, and it has been postulated to be the ancestral P450 isoform [34]. This family is functionally conserved and has a very limited range of substrates related to the biosynthesis of sterols and their derivatives, and a high conservation within the SRSs is a specific feature of CYP51. It was believed that two motifs, "YxxF/L(i)xxPxFGxxVxF/YD/a" and "GQ/hHT/sS", present within the regions of SRS1 and SRS4, respectively, can be considered as the CYP51 signature [35]. In this study, no CYP51-like gene has been identified with such a signature in the *T. thermophila *P450 family. So far, all studied CYP51 family members were found to exhibit catalytic function in the oxidative removal of the 14α-methyl group from sterol precursors formed downstream from cyclization of squalene 2,3-epoxide, and the 14α-demethylated products are intermediates in the sterol biosynthetic pathways leading to formation of cholesterol in animals, ergosterol in fungi and a variety of functional sterols in plants and algae [35,36]. CYP51 appears to be absent in insects and nematodes due to the fact that they don't make cholesterol and lack the post-squalene sterol biosynthesis pathway, and absence of the CYP51 gene was thought to be the consequence of gene loss during evolution [25]. Thus, a similar CYP51 gene loss may be inferred in *T. thermophila*. Instead of the sterols in most other eukaryotes, *Tetrahymena *produces the pentacyclic triterpenoid alcohol 'tetrahymanol' and/or hopanoids *de novo *as functionally equivalent structural components of cell membranes, and its ability to synthesize and use this "primitive" substance can be considered as a metabolic relic [37]. Besides, although *T. thermophila *is able to incorporate exogenous sterols into cell membranes and convert those to various derivates, it utilize enzymes other than P450s in the downstream metabolism of certain steroid compounds, such as the cytochrome b5 in the direct desaturation reaction of cholesterol [38].

**Figure 3:**
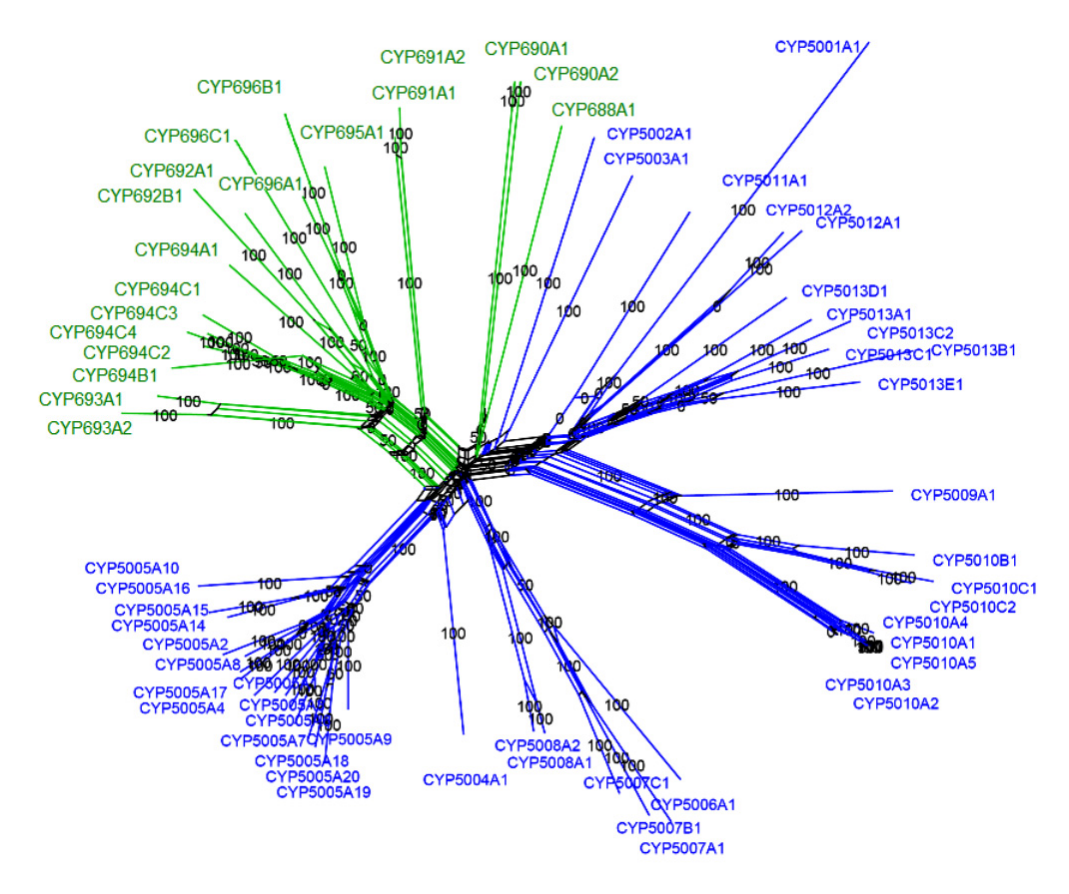
**The phylogenetic network of the *T. thermophila *and *P. tetraurelia *P450 protein sequences**. Green: *P. tetraurelia*; Blue: *T. thermophila*. Bootstrap values are indicated on each node.

The ciliate *P. tetraurelia *is the species closest to *T. thermophila *that has an available genome database and phylogenetic analyses between these two species can help to understand the evolutionary relationship of *T. thermophila *P450 families with other ciliate members. A phylogenetic network was constructed of both the *T. thermophila *and *P. tetraurelia *P450 protein sequences (Figure 3). In the branch that constitutes three *P. tetraurelia *P450 genes (*CYP688A1*, *CYP690A1 *and *CYP690A2*), two genes from *T. thermophila *(*CYP5002A1 *and *CYP5003A1*) also present and cluster together. However, according to the deep branching of these sequences and the relatively low bootstrap values on the branch nodes (50%), there is yet no strong evidence supporting the possibility that they represent orthologs shared by the two species. Surprisingly, the other major clades of P450 genes seem to be highly specific to *T. thermophila *or to *P. tetraurelia*. Moreover, the number of P450 genes in *P. tetraurelia *is less than half of that in *T. thermophila*, and Blast searches in different EST databases revealed that only about 1/3 of the *P. tetraurelia *P450s have retrievable EST data (C. Fu, unpublished observations). It has been pointed out that while *Tetrahymena *and *Paramecium *are related species within the diverse clade of ciliates, their genomes have obviously evolved by significantly different mechanisms [39]. The *Paramecium *genome has gone through a series of whole genome duplications and may exhibit the most completely preserved whole-genome duplication described to date, where ~95% of the genome is located in duplicated blocks [40]. By contrast, although up to 40% of the *T. thermophila *genome was also indicated to be located within duplicated blocks, the most of the large expansions of its gene families may have arisen through tandem duplication events [41]. While differences in the P450 genes in the two species may reflect the difference in evolutionary strategies between the two organisms since their divergence, further investigations are needed to address the adaptive significance of these diversities relative to the well known functions of cytochrome P450s in sensing of and responding to environmental changes, particularly environmental stress.

### P450 gene expression analysis

Spatial and temporal gene expression patterns are important aspects of gene regulation and expression pattern analysis has played an important role in the study of the cytochrome P450 gene superfamily. In the present study, the gene expression patterns of all *T. thermophila *P450s during three important cell physiological stages were analyzed based on EST and microarray data.

To examine the expression profiles of the P450 genes, we initially retrieved the EST data of *T. thermophila *from both in the NCBI EST database and the taxonomically broad EST database (TBestDB) (Apr. 2008) that had been derived from cDNA libraries made from cells in different physiological conditions. The ESTs matching with a corresponding predicted P450 gene are listed in Table 1, which indicates that the gene of origin is expressed and the numbers of ESTs found could be considered as a first indication of the relative expression abundance of that gene. For example, ESTs derived from a few P450 genes (*CYP5005A18*, *CYP5005A19*, *CYP5008A1*, *CYP5010B1*, *CYP5013A1 *and *CYP5013D1*) were found a number of times and their relatively high expression levels were also checked by semi-quantitative RT-PCR analysis, while ESTs from many other P450s were not identified at any of the examined cell physiological/developmental stages. While EST analyses enabled the localization of introns and demonstration of a likely case of alternative splicing, due to random fluctuation in EST numbers and differences between the sizes of each library, the total EST counts in different cDNA libraries cannot be rigorously compared for quantifying the relative amounts of the expressed genes in different physiological states.

**Figure 4:**
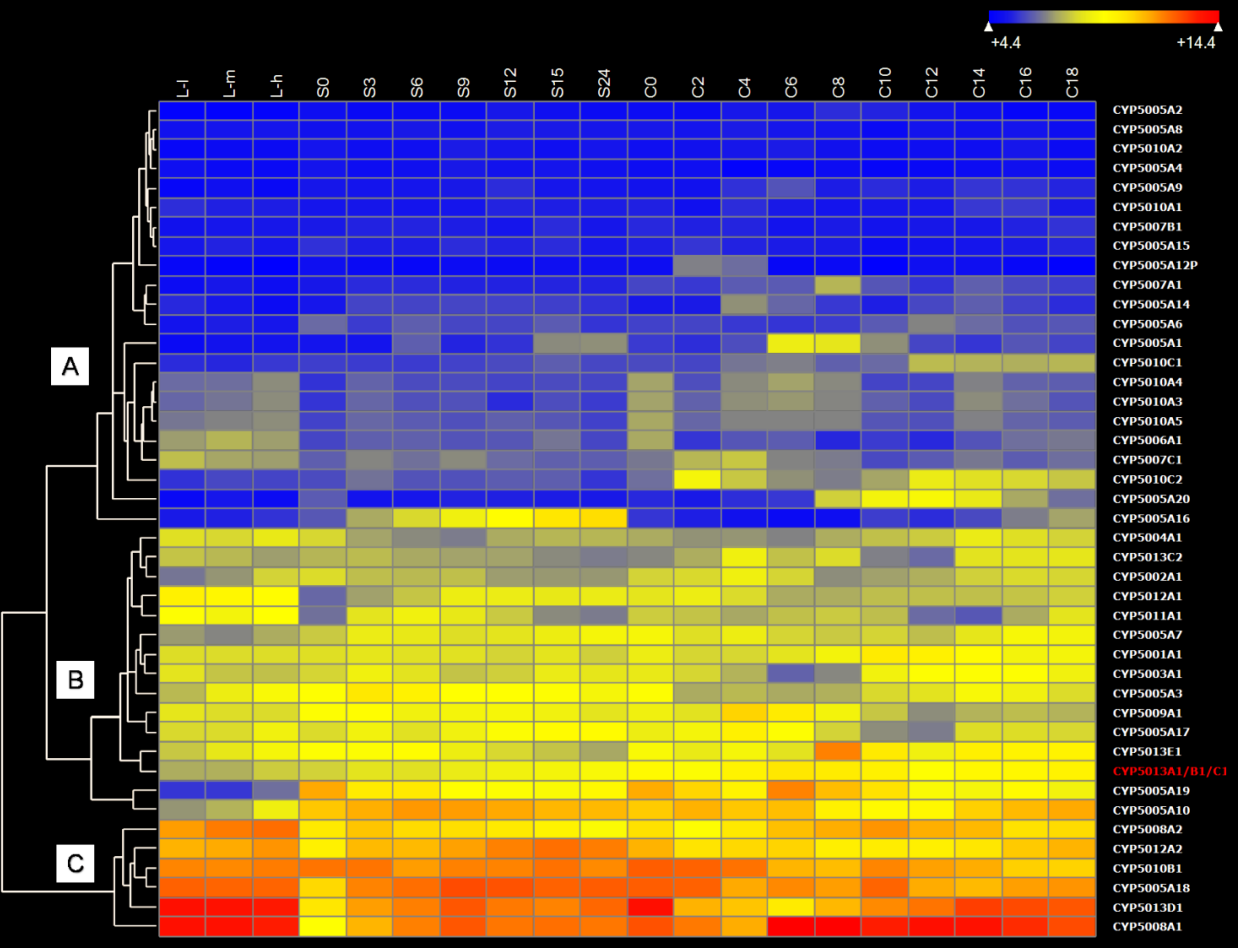
**Heatmap of *T. thermophila *P450 gene expression patterns**. The levels of expression are illustrated by different grades of color scale determined on the basis of the microarray data, as indicated from the top bar (From left to right): Dark color: low expression; light color: high expression. P450 gene names are shown on the y-axis and the three physiological stages examined on the x-axis. The "monster gene" represents the false prediction of merging the three P450 genes (*CYP5013A1*, *CYP5013C1 *and *CYP5013B1*) into one gene by TIGR, resulting in their being combined on the microarray. Vegetative growth: **L-1**, low cell density (100,000 cell/ml); **L-m**, medium density (350,000 cell/ml); **L-h**, high cell density (1,000,000 cell/ml); Starvation: **S0**, 0 h; **S3**, 3 h; **S6**, 6 h; **S9**, 9 h; **S12**, 12 h; **S15**, 15 h; **S24 **24 h; Conjugation: **C0**, 0 h; **C2**, 2 h;**C4**, 4 h; **C6**, 6 h; **C8**, 8 h; **C10**, 10 h; **C12**, 12 h;**C14**, 14 h; **C16**, 16 h; **C18**, 18 h.

Therefore, a more comprehensive analysis of the gene-expression patterns of the each *T. thermophila *P450 family member was done by whole-genome microarray analyses of RNAs from three different cell physiological/developmental conditions (vegetative growth, nutrient starvation and conjugation). Cluster analysis of the heat map of the P450 expression profiles among the different conditions showed all the P450 genes could be clustered into three major clades on the basis of their basal expression levels and dynamic fluctuation patterns during the three different stages. Generally, three main categories of expression patterns could be postulated. The first category contains P450 genes that are silent or have relatively low expression levels during all the experimental conditions and time scales (most genes in clade A in Figure 4). One surprising phenomenon is that most of the P450 isoforms within this group come from branches having many paralogs in the phylogenetic clade, such as the CYP5005 and CYP5010 family. Although these genes are possibly becoming pseudogenes after relatively recent duplication events, the transcriptional activities of these genes at a specific physiological phase other than the three major ones examined here, or under some xenobiotic stimulus cannot yet be excluded.

Secondly, nearly 50% of all the P450 isoforms, including the mostly highly expressed isoforms (*CYP5005A18*, *CYP5008A1*, *CYP5008A2*, *CYP5010B1*, *CYP5012A2 *and *CYP5013D1*) are constantly expressed at all life cycle stages, although their expression levels may vary to different extents (clades B and C in Figure 4). These genes probably take part in constitutive, endogenous physiological processes. It should be pointed out that, due to an erroneous prediction of the TIGR genome sequence that merged three adjacent P450 genes (*CYP5013A1*, *CYP5013C1 *and *CYP5013B1*) into one "monster" gene when the microarray was designed; the separate microarray data for these three genes were unavailable. However, by manually checking the signal of 5 of the 14 oligonucleotide probes whose position correctly matched the putative *CYP5013A1 *gene, its high expression level could be inferred. The EST information of *CYP5013A1 *also supports this conclusion.

**Figure 5:**
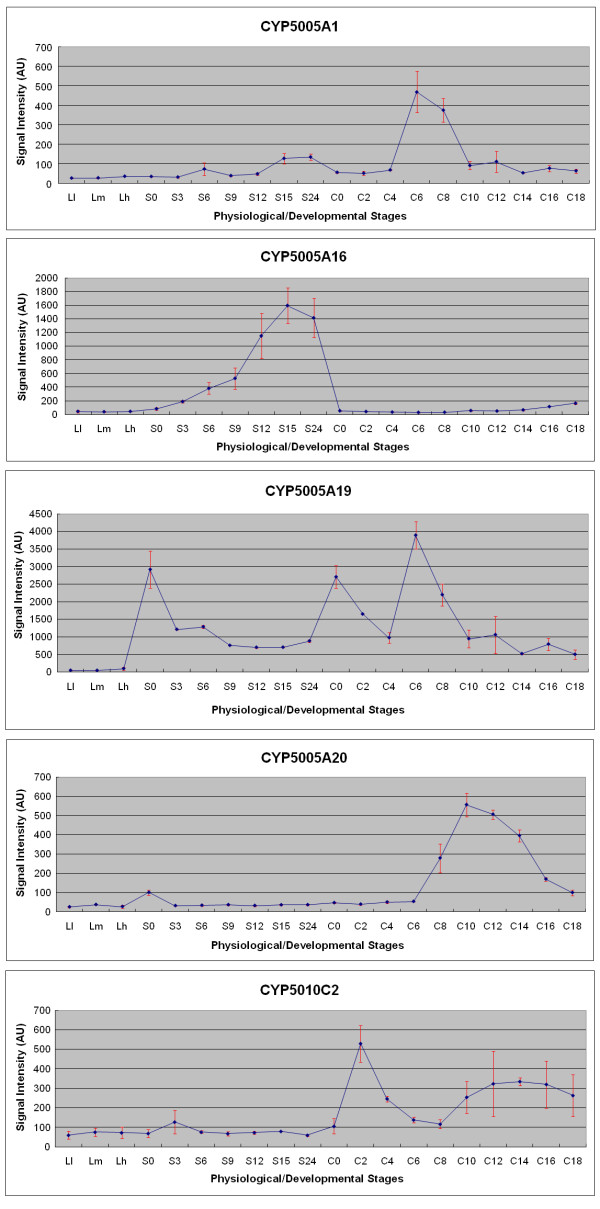
**Gene expression patterns of P450 genes that belong to the third category**. This category consists of P450 genes whose expression levels varied markedly at a specific time period or under a certain physiological condition. Gene relative expression levels are shown on the y-axis and the three examined cell physiological stages on the x-axis. Vegetative growth: (L-l to L-h); Starvation: (S-0 to S-24); Conjugation: (C-0 to C-18).

The last category consists of five P450 genes whose expression levels varied markedly at specific time points or during one of the three physiological conditions that have been examined, including *CYP5005A1*, *CYP5005A16*, *CYP5005A19*, *CYP5005A20 *and *CYP5010C2 *(Figure 5). This probably indicates their involvement in some important, stage-specific endogenous cellular process. Starvation is not only a distinct physiological state that *Tetrahymena *likely encounters in its freshwater environment, but it also induces numerous phenotypic and behavioral changes resulting in the acquisition of competence for mating [42]; The nuclear events that occur in conjugating *Tetrahymena *have clear parallels in multicellular eukaryotes and include meiosis, pronuclear formation, pronuclear fusion, postzygotic divisions, and cytoplasmic determination of nuclear fate [43,44]. Thus, further studies are necessary to correlate the expression level of specific P450 isoforms with stage-specific cellular processes.

Meanwhile, expression studies of *T. thermophila *cytochrome P450 genes when exposed to specific chemical substances are currently under investigation. Among the P450 genes that are not expressed in the three major physiological/developmental states, at least three of these isoforms (*CYP5007C1*, *CYP5010A4 *and *CYP5010A5*) are transcriptionally active under different xenobiotic stresses (W. Miao and C. Fu, unpublished microarray data), suggesting that they may play a role in the metabolism and biotransformation of some chemical compounds.

### Evolutionary analysis of *T. thermophila *P450 genes

#### Codon-usage analysis

**Figure 6:**
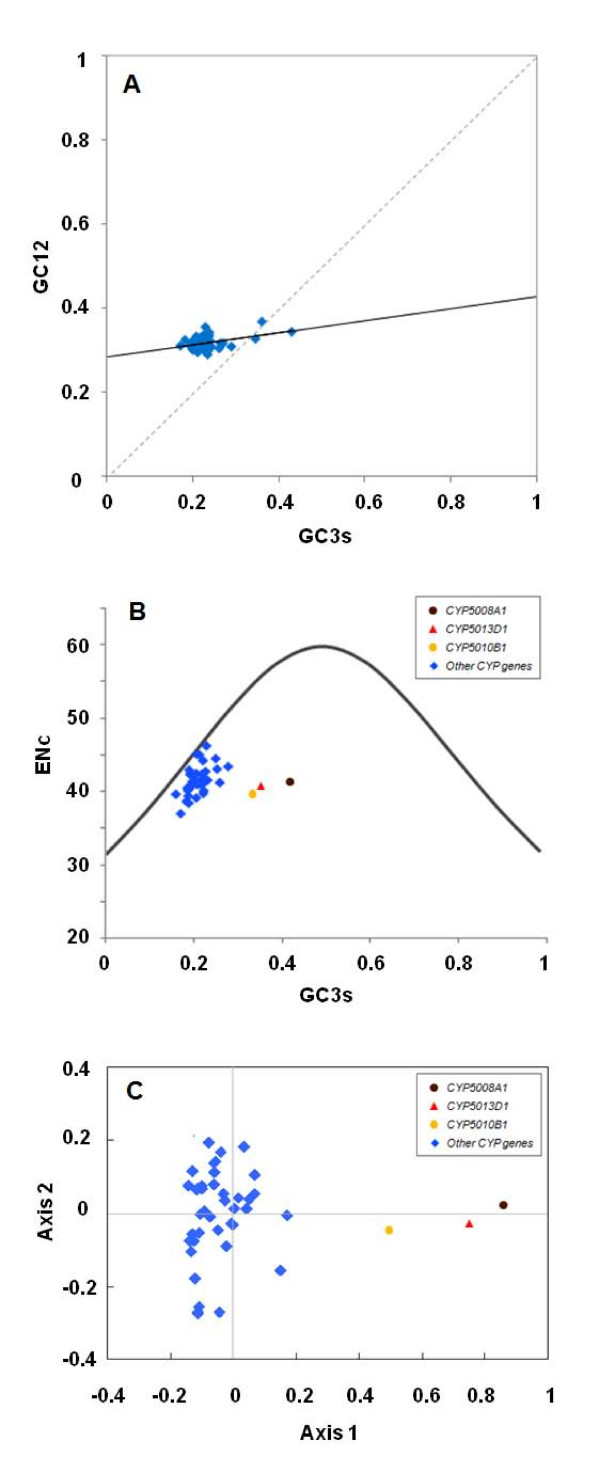
**Codon usage in *T. thermophila *P450 genes**. A: Neutrality plots (GC12 vs. GC3s). The regression line: y = 0.1435x + 0.2853, r2 = 0.1877, OP (optimal point) = 0.333. OP indicates the point at which the regression line crossed the diagonal line. B: The Effective number of codons (ENc) plotted with the GC3s for each predicted P450 gene. Each expected ENc from GC3s is shown as a standard curve. C: Correspondence analysis of the relative synonymous codon usage (RSCU) values. A plot of the two most important axes after the correspondence analysis was shown. The three P450 isoforms (*CYP5008A1*, *CYP5010B1 *and *CYP5013D1*) that each has a CAI value above 0.6 were indicated with brown, orange and red color dot, respectively. The rest of the *T. thermophila *P450 genes were shown with a blue dot.

Codon usage bias in genes is an important evolutionary phenomenon and has been widely examined among prokaryotic and eukaryotic organisms. Firstly, since the codon bias can be shaped by preferences at the level of nucleotide, specifically the GC content levels of the coding regions, we carried out a relative neutrality plot analysis (GC12 plotting against GC3s) of the *T. thermophila *P450 genes (Figure 6A). Unlike GC3s, GC1 and GC2 are subject to functional constraints against change because a mutation at these positions usually leads to an amino acid change, except between some codons of arginine, leucine, or serine [45]. The neutrality plot of the *T. thermophila *P450 genes showed that there is a significantly positive correlation (p < 0.01) and the correlation coefficient was 0.433, indicating that the effect on the GC contents by the intragenic GC mutation bias was similar at all three codon positions. Since both the neutral mutation and selective constraint play roles in shaping codon usage pattern of gene sequences [45], this suggests that there were relatively high mutation biases or low conservation of GC content levels among the *T. thermophila *P450 genes.

Secondly, if translational selection pressure influences the shaping of codon usage, the bias would show significant positive correlation to expression levels and some translation-preferred codons should be used more frequently than others. Thus, we calculated the two often used measures of codon usage bias, the CAI [46] and the ENc [47] of each *T. thermophila *P450 gene,. Among the most highly expressed P450 genes (clade C in Figure 4), three isoforms (*CYP5008A1*, *CYP5010B1 *and *CYP5013D1*) have a CAI value above 0.6, which is significantly above that of the average (0.421) following a student's t test (p < 0.05). Meanwhile, four P450 genes (*CYP5005A18*, *CYP5008A2*, *CYP5012A2 *and *CYP5013A1*), although exhibiting a similar high basal expression level, showed a CAI value close to, or even less than, that of the average. Thus, no simple trend exists between the expression levels of P450 genes and the codons they use. Meanwhile, in the ENc-plot analysis, if a given gene is only subject to G+C composition mutation constraint, it will lie above or just below the standard curve. From Figure 6B, it can be seen that while the most *T. thermophila *P450 genes had ENc values slightly smaller than expected ones, the three genes with the significant high CAI values displayed a more biased codon usage according to the respective GC3s, indicating that they are probably under pressure from direct expression selection.

The correspondence analysis on the RSCU was also performed to avoid identification of trends in codon usage due to biased amino acid usage among the genes. RSCU of *T. thermophila CYP *genes detected one major trend on the first axis of inertia which accounted for 26.05% of the total variation and was approximately three times as much of the variation as the second axis (8.87%), and no other axes accounted for more than 7.34% of the total variation, respectively. The codons of *T. thermophila CYP *genes that make the major contributors to this pattern showed preference for ending in C, including CCC (Proline), UCC (Serine), CUC (Leucine), GUC (Valine), CAC (Histidine) and AUC (Isoleucine), and this might indicate that codons ending in C could lead to better translational accuracy/efficiency. Besides, Axis 1 was significantly correlated to GC and GC3s (r = 0.913, 0.931, *p *< 0.01, Pearson correlation coefficient), but not to ENc (r = 0.074, *p *> 0.05). While this result implies that the nucleotide composition mutation could be considered as the relatively major factor in shaping the codon usage in P450 genes, it should be taken into account that the robustness of our current analysis would be limited by the relative small quantities of calculated genes. A recent study reported that there was a very small but significant correlation between the ENc and expression level (estimated based on the EST counts) in *T. thermophila *[48]. However, in Miao et al. [20] the authors found that while most codon-biased genes (95%) are expressed constitutively and at high levels, only a fraction of highly expressed genes (<15%) are codon-biased. This may well reflect that the genome-wide microarray analyses covered a wide range of physiological/developmental stages and are subject to less bias than non-saturated, random analyses of cDNAs. Thus, although the overall codon usage biased is determined by nucleotide composition mutation in *T. thermophila*, the presence of a preferred gene subset is under pressure from direct expression selection which probably results from the large effective population size of this species [49]. It has been reported that in the bacteria *Bacillus subtilis *[50] and *Chlamydia trachomatis *[51], the synonymous codon usage appeared to be determined by both the neutral mutational biases and translation selection. Therefore, while genes within one species often share a single codon usage pattern, exceptions also exist, especially among microorganisms.

#### Site-specific selection of duplicate P450 genes

The two largest P450 families in *T. thermophila *(CYP5005 and CYP5010) were chosen for investigating whether different evolutionary pressures exist on particular sites of P450 isoforms. Here the rates of substitutions that occurred within the coding regions of the family members were calculated for all types of selection (purifying, neutral and positive). Different rates of substitutions were estimated to occur within the coding regions among the two family members. The obtained Ka/Ks ratios were between low (<1) to median (= 1) along the whole sequences, and were especially low among some residues that represent some core P450 structure regions, indicating strong negative selection on these structural regions. This suggests they are functionally important sites in catalytic reactions and may experience restriction of deleterious mutations. Meanwhile, no positive selection was indicated at any specific site along the rest of gene coding regions, suggesting a divergent or neutral evolution process among both the CYP5005 and CYP5010 family members (Additional file 5).

In ciliate species, fast protein evolution has been reported for several genes, and was suggested to be the consequence of either relaxed functional constraint on the nucleosomes of amitotic macronuclei or of adaptive evolution through gene duplication coupled with the ciliates' highly processed macronuclear genomes [52]. The low expression levels of many isoforms within the CYP5005 family, suggested they might have undergone pseudogene formation. However, the possibility cannot be ruled out that some members of these groups serve as inducible P450 isoforms that function when the cell contacts specific xenobiotic compounds from the environment. In a recent comparative genome analysis of vertebrate cytochrome P450 genes [12], it was suggested that all of the CYP genes that encode enzymes with known endogenous substrates are phylogenetically stable, characterized by few or no gene duplications or losses. In contrast, the unstable P450 genes that are characterized by frequent gene duplications and losses were found to constitute most of those that encode enzymes that function as xenobiotic detoxifiers. Besides, adaptive evolution has also been estimated to occur restrictively on those duplicated CYP450 genes at the amino acid sites within the SRSs regions in the protein structure. This may be due to their functional association with unstable environmental interactions such as toxin and pathogen exposure. Such specific xenobiotic-driven P450 gene expansion events were also observed in *D. melanogaster *[53] and *C. elegans *[54]. The relatively high heterogeneity among the major clades within the *T. thermophila *P450 family members, along with their diverse expression patterns suggested that a similar situation might be occurring in this organism.

#### Expression divergence of duplicate P450 genes

In evolving genomes, change in gene expression is one important mechanism that can lead to retention of duplicate genes, and studying the gene expression patterns has been suggested to be an important measure of gene functions that can facilitate our understanding of the genetic basis of evolutionary change [55,56]. High-throughput approaches, such as microarray techniques, provide an opportunity to investigate gene expression of whole genomes simultaneously, allowing studies of how different genes respond to a certain environmental stimuli and the general gene expression patterns among various gene families that were categorized into different cellular functions on genome-wide scales [57].

We first investigated the gene-expression evolution patterns of the P450 duplicate pairs in the two largest *T. thermophila *P450 gene families, CYP5005 and CYP5010, by analyzing the gene-expression data from multiple microarray experiments. For the three physiological/developmental stages, the *T. thermophila *cells examined, the nonphylogenetic model was the best supported model both for the two families (Additional file 6). For the CYP5005 family, the best "nonphylogenetic-free" model suggested that more closely related duplicate genes are no more likely than more distantly related genes to share similar expression patterns. This may indicate either the gene family has little influence over physiological functions or the rapid rates of gene-expression evolution [55]. Meanwhile, the "nonphylogenetic-distance" model best fit the CYP5010 family, indicating that genetic distances since last gene duplication predict change in expression, consistent with an initial coupling during evolution of expression and coding sequences, i.e. a correlation between the genetic distance in the coding region of CYP5010 family members and the change in gene expression level still can be detected (Additional file 7). This was similar to a report on duplicate genes in the yeast *S. cerevisiae *showing that gene expression patterns remain similar shortly after gene duplication, but the evolution of expression occurs quickly so that the patterns become distinct from each other in a relatively short period of time [58]. Due to the observation that many CYP5005 family members are within the "expression-silent" category, the assumed gene pseudonization events probably obscured the possible correlation between the genetic distance and gene-expression divergence in the CYP5005 family.

Further, we estimated the relative duplication time of the two gene families. Since silent substitution rates were often used as an approximation to the neutral mutation rates [59], we calculated the 4-fold substitution rates (d4) of the synonymous sites for all the duplicate pairs for each of the two gene families. It was indicated that except for three genes (*CYP5010A3*, *CYP5010A4 *and *CYP5010A5*, d4<0.015) that raised by very recent duplication events (Figure 1), most P450 genes in the CYP5005 and CYP5010 families have uniform mutation rates (1.072 and 0.880, on average). Then we calculated the amino acid distance between duplicates for using as a proxy for evolutionary time. The results showed that the overall distance values are highly identical (0.748 and 0.742, on average), thus indicating that the duplication time of the two gene families was close to each other. Therefore, given that the enlargement of these P450 families in *T. thermophila *was probably caused by recent separate small duplication events, the divergence rate of gene expression thus may also vary between the two P450 gene families.

Since a rapid divergence of expression among the *T. thermophila *duplicate P450 genes could be inferred, we also tried to examine the 5' flanking regions of the *T. thermophila *duplicate P450 genes for identification of likely regulatory elements. It is well known that promoters and enhancers located upstream of the coding regions usually have critical roles in regulating gene expression levels, and that major P450 genes are selectively induced by different nuclear receptors in response to endogenous substances or diverse xenochemicals in multicellular organisms [60,61]. However, the fact that the *Tetrahymena *genome has a 78% A/T content makes it difficult to identify regulatory elements by sequence homology to well-characterized promoters from other model organisms using transcriptional regulation databases such as TRANSFAC [62]. Currently, except for a few regulatory elements that have been identified by deletion and mutational analysis [63,64], little information is available on the *cis*-elements of specific genes in *Tetrahymena*. We further checked possibility whether the *T. thermophila *P450 duplicate pairs or their adjacent genes in the same chromosome (scaffold) have a tendency to show similar physiological/stage-specific patterns of expression. The results showed that most of these genes exhibited wide variations in their levels and stage-specificity of expression, i.e. no chromosomal or sub-chromosomal level of gene regulation was indicated (data not shown). Therefore, although the rapid divergence of upstream non-coding sequences of the relatively well-conserved P450 OFRs may contribute to the remarkably varied expression patterns among the *T. thermophila *duplicate P450 genes, further experimental investigations are needed to identify specific regulatory elements or *trans*-acting factors involved in the transcriptional induction of P450s in this organism.

## Conclusion

In the current study, we identified 44 putative functional P450 monooxygenase genes in the model ciliate organism *T. thermophila*, analyzed their evolutionary relationships and characterized their expression based on both EST and microarray data, using bioinformatics tools. The current microarray data provide background information of *T. thermophila *P450 gene expression in normal physiological states. Our analyses provide information on the characteristics and diversity of the P450 genes in the Ciliophora, and will facilitate further functional analysis to understand the roles of individual P450 isoforms either in cellular physiological metabolism or the possible oxidative detoxification catalysis under environmental toxic exposures in this model ciliate species.

## Methods

### *T. thermophila *putative P450 Gene identification and cDNA cloning

Putative *T. thermophila *cytochrome P450 gene sequences were retrieved from the Institute of Genome Research (TIGR; now known as the J. Craig Venter Institute) predicted peptide database (Preliminary_Gene_Predictions_Aug_2004.pep), used to search against both the *T. thermophila *genome sequences database from TIGR (Assembly2-Nov_2003.scaffolds) and the TGD (Tetrahymena Genome Database, ) [65] predicted gene database using Blastp with the filter option turned off. The P450 gene predictions made by Dr. David Nelson were used as an independent source (available at ). The deduced amino acid sequences obtained from the above two strategies were then compared. To improve the accuracy of exon and signal peptide identification in the putative genes, the canonical GT/AG rule was used to determine the intron splice sites and all corresponding P450 nucleotide sequences were subjected to BLAST N searches against both the NCBI EST database  and the Protist EST Program (TBestDB, ) [66]. For some putative genes with uncertain intron boundaries, RT-PCR was carried out to check the cDNA sequences. Total RNA of 2 ml cultures of *T. thermophila *exponential phase cells (strain SB210) was isolated by the TRIzol reagent (Gibco BRL) method according to the user manual with slight modification. RNA purity and integrity were monitored by spectrophotometry and electrophoresis. RNA samples were treated with DNase I (Promega) and reverse transcribed into double strand cDNA using MMLV (Promega) in a 25 μL reaction mix. Primers (Additional file 8) were designed using Primer3 . PCR products were sub-cloned into the vector pGEM-T (Promega, Madison, USA), transformed into *Escherichia coli *DH5α and sequenced using the ABI model 377 stretch automated DNA sequencer (PE Applied Biosystems). A pair of primers (5013A1Int-Fw, 5013A1Int-Re) that span the intron junctions was used to check the alternative splicing of the *CYP5013A1 *intron, and the different transcripts were further investigated by RT-PCR experiments coupled with non-denaturing PAGE electrophoresis.

### Sequence alignment and phylogenetic analysis

All predicted P450 genes except pseudogene sequences from *T. thermophila *were used for alignment and phylogenetic analysis at the amino acid level. The pairwise levels of gene similarity/identity were calculated using the program MegAlign, which is embedded in the DNASTAR package (DNASTAR, Inc.) [67]. Multiple sequence alignment (MSA) of the P450 proteins was conducted using both the CLUSTALW program at the EMBL-EBI website  and the T-coffee (Tree-based Consistency Objective Function For alignment Evaluation) server , using parameters under default settings. The quality of each resulting alignment was evaluated by the CORE method, available through the T-coffee server, compared and manually improved by removing any badly aligned columns. For construction of the phylogenetic tree, the Neighbor-Joining (NJ) method (JTT matrix with different rates among sites, gamma parameter = 1.0, bootstrap test = 1000 replicates) was applied on the MSA using MEGA (version 4.0) [68]. In addition, a maximum-likelihood (ML) tree was constructed with PhyML [69] (JTT matrix, four rate categories, gamma distribution parameter = estimated). The resulting tree was tested with 200 bootstrap repeats with PhyML with the same settings. Putative full length P450 genes of *Paramecium tetraurelia *were retrieved from (ParameciumDB [70] and the P450 website , respectively. Totally, 19 *P. tetraurelia *P450 genes that either were consistent with the available EST data or shown to be identical in the above two predictions were chosen for the following analysis, and the standard nomenclature was used according to Nelson's website. MSA of both the *T. thermophila and P. tetraurelia *P450 protein sequences was conducted using the T-coffee program and manually improved and a maximum-likelihood tree was constructed by PhyML program. Phylogenetic network was conducted with the NeighborNet method using the SplitsTree program (version 4.10) [71], (Model = JTT, chartransform = ProteinMLdist, splitstransform = EqualAngle, gamma distribution parameter = 2.0, bootstrap test = 1000 replicates)

### Secondary structure elements assignment and sub-cellular localization predictions

One *T. thermophila *P450 sequence (*CYP5002A1*, *CYP5005A18*, *CYP5007C1*, *CYP5008A1*, *CYP5011A1*, *CYP5012A1 *and *CYP5013A1*) from each of seven family clades and one *P. tetraurelia *P450 sequence (*CYP693A1*, ParameciumDB accession No. GSPATP00036495001) that have full length or partial cDNA information were selected as the representative set. Previously reported P450 protein crystal structure data were downloaded from the RCSB (Brookhaven protein data bank) website , including P450cam (CYP101), P450BM3 (CYP102), P450terp (CYP108) and P450eryF (CYP107A1) from bacteria, P450nor (CYP55A1) from fungi, CYP2C5, CYP2C8 and CYP3A4 from mammals, corresponding to PDB codes 1UTU, 1ZO4, 1CPT, 1EUP, 1EHE, 1NR6, 1PQ2 and 1TQN, respectively. With each of the indicated P450 proteins, MSAs were done at the amino acid level through the 3DCoffee web server , which affords the possibility of producing an MSA with combined sequence and structure information. Judging by sequence identity from all the examined alignment results and CORE index, mammalian P450 CYP3A4 was chosen as the template for secondary structure. The program ESPript [72] was used for the assignment of secondary structure elements onto the corresponding aligned sequences, and substrate recognition sites were manually indicated based on the CYP3A4 enzyme information. Then obtained aligned sequences were used to investigate the conservation pattern of the *T. thermophila *P450 family as inferred by Consurf program [73]. All putative functional *T. thermophila *P450 proteins were checked for likely sub-cellular localization by using the TargetP program [74] and the WoLF PSORT [75] with the default parameters.

### Cell culture, RNA extraction and Semi-quantitative RT-PCR

Cells in the growth, starvation (strain SB210) or conjugation (strains CU428 and B2086) stages of the life cycle were used for RNA extraction. Semi-quantitative RT-PCR analysis using gene-specific primers for selected P450 genes were carried out. A pair of primers designed for the *T. thermophila *β-actin gene (Genbank accession No. AY315823) was used as the control for normalization of expression data. PCR cycling conditions were: 5 min at 95°C; 20 s at 94°C; 20 s at 60°C; 60 s at 72°C in a 25 μL reaction with totally 30 cycles. To calculate the normalized relative gene expression levels, the same amount of PCR products underwent a 1% EB stained agarose gel electrophoresis and scanned pictures were taken. The relative expression levels were calculated by lane analysis using the QuantiScan for Windows (Biosoft, Cambridge, England) software according to the tutorial.

### Analyses of microarray data

RNA sample preparation, *Tetrahymena thermophila *whole-genome oligonucleotide DNA Microarray synthesis and data analysis are described in Miao et al. [20]. In brief, wild-type cell lines B2086 and CU428 of *T. thermophila *were provided by Dr. P.J. Bruns, Cornell University, Ithaca, NY. Both of these cell lines have inbred strain B genetic background, as does cell line SB210, the source of the MAC genome sequence used to design the microarray probes. Cells were grown in SPP medium [76] at 30°C. For microarray analyses of growing cells, CU428 cells at low, medium and high cell densities (~1 × 10^5 ^cells/ml, ~3.5 × 10^5 ^cells/ml and ~1 × 10^6 ^cells/ml; referred to as L-l, L-m and L-h) were collected. For starvation, CU428 cells at 2 × 10^5 ^cells/ml were washed and starved at 2 × 10^5 ^cells/ml in 10 mM Tris (pH 7.5); samples were collected at 3, 6, 9, 12, 15 and 24 hours referred to as S-0, S-3, S-6, S-9, S-12, S-15 and S-24). For conjugation, equal volumes of B2086 and CU428 cells that had been starved for 18 hours in 10 mM Tris (pH 7.5) at 2 × 10^5 ^cells/ml, were mixed, and samples were collected at 0, 2, 4, 6, 8, 10, 12, 14, 16 and 18 hours after mixing (referred to as C-0, C-2, C-4, C-6, C-8, C-10, C-12, C-14, C-16 and C-18). Total RNA was extracted using the RNeasy Protect Cell Mini Kit (Qiagen, Valenica, CA) according to manufacturer's instructions. cDNA synthesis and Cy3 labeling was performed by NimbleGen Systems, Inc. The custom microarrays were manufactured by NimbleGen Systems, Inc. using the maskless photolithography method described previously [77]. For each growing and starved *Tetrahymena *sample, hybridizations were performed on three independent microarrays. For analysis of conjugation, hybridizations were performed on two independent microarrays. The final data were analyzed based on the RMA-processed expression values (RMA calls). The *r*^2^, fold changes, *p *values and heat maps were calculated using ArrayStar software, version 2.0 (DNASTAR, Inc, Madison, WI).

### Codon-usage analysis

G+C contents of each entire gene, first and second, third codon positions (GC, GC1, GC2 and GC3s, respectively) were calculated for each *T. thermophila *P450 gene using the CodonW software  and DnaSP4.0 [78]. GC12 was the average of GC1 and GC2 and was used for neutrality plot analysis. The strength of the selection on a given gene relative to the mutation pressure were estimated by the method of the relative neutrality plot (RNP), which consists of plotting the G+C content at the nonsynonymous positions (GC12) of the codons against the G+C content at the synonymous position (GC3s). If GC12 was as neutral as GC3s against selection, the points should be distributed along the diagonal line (slope of unity). In contrast, if GC12 was completely non-neutral, the points should be on the parallel lines of abscissa (slope of zero). Thus, the regression coefficient (slope) provided a measure of relative neutrality of GC12 to GC3s.

The CAI and the ENc values were both calculated of each *T. thermophila *P450 genes, respectively. The CAI indicates the similarity of a gene in its codon usage pattern compared to that of a predefined gene set in the same organism. While the *T. thermophila *genome is very AT rich with a bias against GC rich codons, a subset of 'preferred' codons that are independent of the genes' AT content and differed from that of the average gene was found to be used with high frequency in a group of 232 highly expressed genes [11], and were used as the reference dataset in the CAI calculation. ENc is a measure of the effective number of codons used in a gene. For the universal genetic code, ENc delivers values that range from 61 (no bias – all codons used equally) to 20 (complete bias – only one codon used for each amino acid). In the case of ciliates, ENc values range from 63 to 20 theoretically, since there are 63 sense codons in their alternate genetic codons. The expected ENc values from GC3s under H0 (null hypothesis, i.e., no selection) were calculated according to Equation 1, where S denoted GC3s [47]:

(1)

Correspondence analysis was used to investigate the major trend in relative synonymous codon usage variation among the genes, using the CodonW software. The RSCU value for a codon is the observed frequency divided by the frequency expected if all synonyms codons for that amino acid were used equally. Only those codons for which there is a synonymous alternative were used in the analysis. Each gene is described by a vector of 59 variables (codons). Correspondence analysis identifies the major trends in the variation of the synonymous codon usage data and distributes genes along continuous axes in accordance with these trends. RSCU values close to 1 indicate a lack of bias, while much higher and much lower values indicate preference and avoidance of that particular codon, respectively. The calculated codon usage parameters of 44 *T. thermophila *P450 genes were listed in Additional file 9. Correlation analysis was performed using SPSS version 12.0 and Microsoft Excel.

### Site-specific selection analysis

Since recombination may result in higher rates of false positives in maximum likelihood tests for positive selection, the possibility of intergenic recombination events in two datasets (CYP5005 and CYP5010 family) were checked using the TOPALi software [79], with the DSS (Difference of Sums of Squares) and PDM (Probabilistic Divergence Measures) methods (window size 100, step 2), and no positive signal was detected. Then the two datasets were selected to detect site-specific positive selection and purifying selection. For each set, an amino acid alignment was conducted using the CLUSTAL W program with default settings. The resulting alignment was used to generate the corresponding codon alignment with RevTrans [80], and to construct an unrooted ML tree with PhyML. The codon alignment and the phylogenetic tree were used for calculation of the ratio (ω) between non-synonymous Ka and synonymous Ks at each site of the predicted protein for all types of selection (purifying, neutral and positive) with Selecton (version 2.4) , according to Stern et al. [81]. The positive selection of the two gene family members were also checked using Likelihood ratio tests (LRTs) with PAML (version 4) [82]

### Gene-expression evolution analysis

Gene-expression evolution analysis was done according to [55]. Firstly, the amino acid sequences of CYP5005 and CYP5010 family members were aligned respectively using the CLUSTAL W program with default settings and translated to the corresponding codon alignment. The (GTR+I+G) model of evolution was selected as determined by likelihood ratio tests in ModelTest [83] and the phylogenetic tree was constructed by ML analysis, using PAUP* version 4.0b10 [84]. The microarray data for each gene at different time points were log-based-two transformed and represent the character data at the taxa tips (see Additional file 7). Then both the phylogenetic tree and the transformed microarray data as a set of continuous data were read into the program CoMET (Continuous-character Model Evaluation and Testing) [85]. The likelihood values for each of the two datasets were calculated using the default punctuation asymmetry threshold of 100. The nine maximum-likelihood models of gene-expression evolution were compared by the Akaike Information Criterion (AIC) and the best fitting evolutionary model was determined by selecting the one with the minimum AIC value. The 4-fold substitution rates (d4), i.e. the expected number of substitutions per site at the four-fold degenerate sites of the third codon position, were calculated as a measuring of the neutral mutation rates using Kumar method in the MEGA program (version 4.0) [68]. The pairwise levels of amino acid distance between duplicate genes were calculated using the JTT Model.

### Nucleotide sequence and microarray data accession numbers

The sequences reported here were deposited in GenBank (Accession No. EU349017–EU349060) at the National Center for Biotechnology Information. Microarray data have been deposited with the NCBI Gene Expression Omnibus  under accession numbers listed in document S11 in Miao et al. [20]. (doi:10.1371/journal.pone.0004429)

## Abbreviations

CAI: Codon Adaptation Index; CORE: consistency of overall residue evaluation; DDT: dichlorodiphenyltrichloroethane; ENc: Effective Number of Codons; ER: endoplasmic reticulum; EST: Expressed Sequence Tag; MSA: multiple sequence alignment; Myr: million years; NMD: nonsense-mediated mRNA decay; ORF: open reading frame; RNP: relative neutrality plot; RSCU: relative synonymous codon usage; RT-PCR: reverse transcriptase polymerase chain reaction; SRSs: substrate recognition sites; UIP: unique intron position.

## Authors' contributions

WM and CF conceived and designed the experiments. CF analyzed the data and performed all experiments except the microarray. WM performed the microarray experiments. JX assisted in analyzing the data and preparing the manuscript. CF and WM interpreted the data and wrote the paper.

## Supplementary Material

Additional file 1 **The position and orientation of three adjacent P450 genes (*CYP5013A1*, *CYP5013C1 *and *CYP5013B1*) in the *T. thermophila *genome**. These three gene isoforms are tandemly located on scaffold 8254607. They were mistakenly merged into one "monster" gene by the TIGR gene finder as was shown in the Genome Browser map. Underline: the putative ORF of *CYP5013A1 *gene. Red italic: the first intron of *CYP5013A1 *gene. Green: the second intron of *CYP5013A1 *gene. Click here for file

Additional file 2 **Sequence alignment of the *CYP5005A7 *gene, ESTs, the pseudogene *CYP5005A5P *and the erroneous pseudogene *TIGR_ CYP5005A7P***. The *CYP5005A7 *gene sequence was obtained from the 2.1kb genomic DNA sequencing results. The two ESTs (TTL00012823 and EC269404) were retrieved from TBestDB and GenBank, respectively. The erroneous sites in the "*TIGR_CYP5005A7P*" sequence were indicated by red squares. The different sites between the *CYP5005A7 *gene and the pseudogene *CYP5005A5P *sequences were indicated by green squares. Click here for file

Additional file 3 **The unrooted maximum-likelihood (ML) tree of the *T. thermophila *P450 protein sequences**. The resulting tree was tested with 200 bootstrap repeats with PhyML and the bootstrap values are indicated on each node. Click here for file

Additional file 4 **The conservation pattern of the *T. thermophila *P450 family**. A: The full indication of the conservation pattern inferred by Consurf based on the multiple sequence alignment using mammalian P450 CYP3A4 as the template of secondary structure elements assignment. Bottom: key to the Consurf colours; B: The six putative substrate recognition sites (SRSs) region were indicated. Click here for file

Additional file 5 **Site-specific selection results of the CYP5005 and CYP5010 gene families**. A: CYP5005 Family; B: CYP5010 Family. A seven-color scale was used by the Selecton program to represent different types of selection. Shades of yellow (colors 1 and 2) indicate ω > 1. Shades of white through magenta (colors 3 through 7) indicate various level of ω ≤ 1. Click here for file

Additional file 6 **Calculated values of the CYP5005 and CYP5010 gene families for each of nine different models**. Calculated Akaike Information Criterion (AIC) and maximum-likelihood (ML) values of the CYP5005 and CYP5010 gene families for each of nine different models were listed. The results of best supported models under each cell conditions were marked in bold. Click here for file

Additional file 7 **Phylogenetic tree of the CYP5010 gene family and its expression profiles for the three physiological/developmental stages of the *T. thermophila *cells**. Left: The ML tree of CYP5010 family used in the gene-expression evolution analysis. Relative branch lengths are proportional to number of substitutions per site. Right: The corresponding log2 transformed microarray data during three cellular conditions as a set of continuous data represent the character data at the taxa tips. Click here for file

Additional file 8 **Oligonucleotide primers used in this study**. Click here for file

Additional file 9 **Codon usage parameters of 44 *T. thermophila *P450 genes**. The CAI, ENc, GC content, GC12, GC3s and the RSCU values of the two most important axes were listed. Click here for file
