# Supplementary figures and images for: Genome-wide identification and characterization of cytochrome P450 monooxygenase genes in the ciliate Tetrahymena thermophila
Source: BMC Genomics. 2009 May 1;10:208. doi: 10.1186/1471-2164-10-208 (PMC2691746; doi:10.1186/1471-2164-10-208)

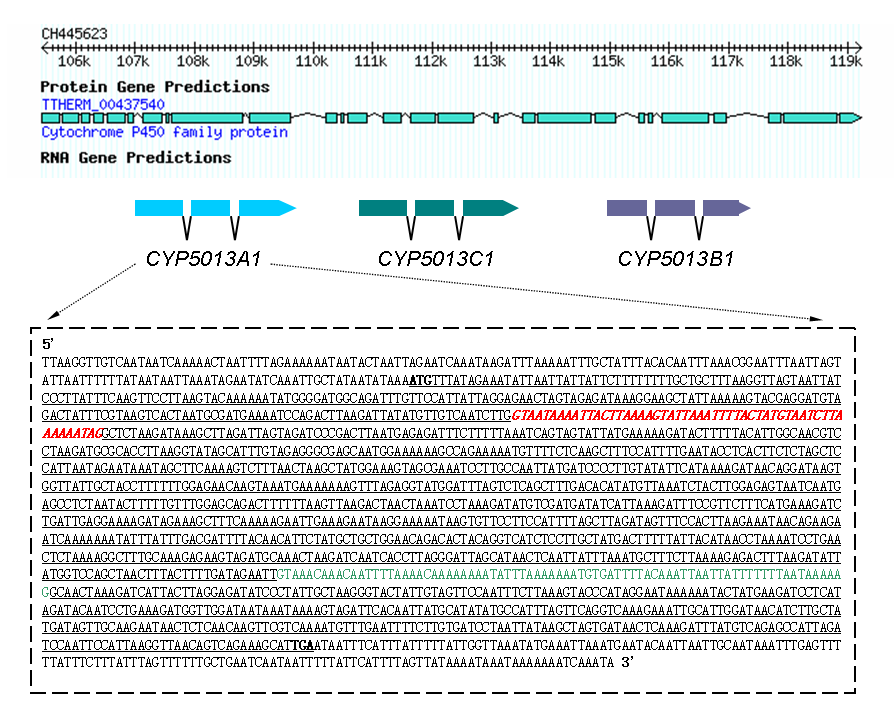

Supplement: Additional file 1 — The position and orientation of three adjacent P450 genes (CYP5013A1, CYP5013C1 and CYP5013B1) in the T. thermophila genome. These three gene isoforms are tandemly located on scaffold 8254607. They were mistakenly merged into one "monster" gene by the TIGR gene finder as was shown in the Genome Browser map. Underline: the putative ORF of CYP5013A1 gene. Red italic: the first intron of CYP5013A1 gene. Green: the second intron of CYP5013A1 gene. [file 1471-2164-10-208-S1.tiff]

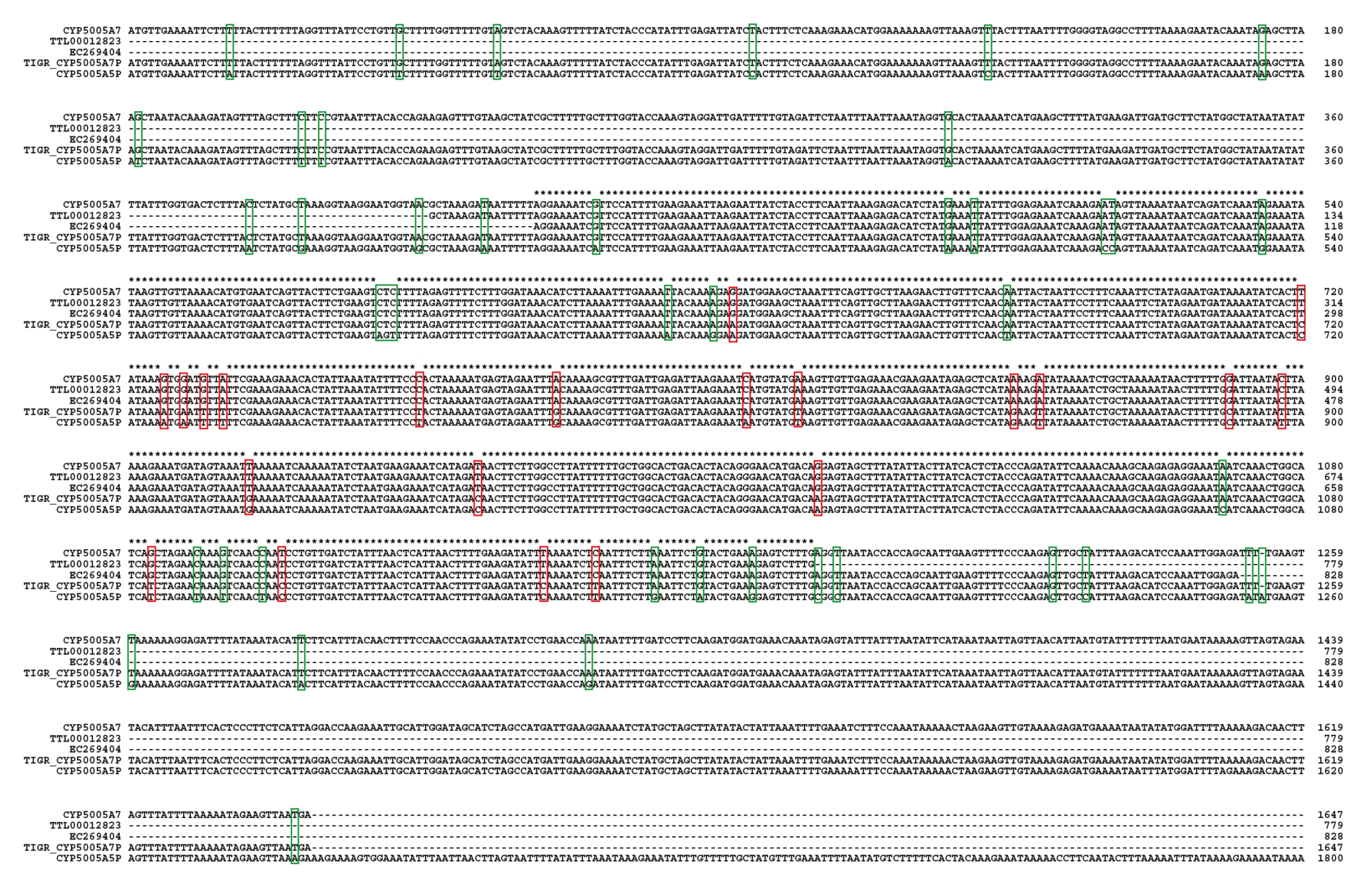

Supplement: Additional file 2 — Sequence alignment of the CYP5005A7 gene, ESTs, the pseudogene CYP5005A5P and the erroneous pseudogene TIGR_ CYP5005A7P. The CYP5005A7 gene sequence was obtained from the 2.1kb genomic DNA sequencing results. The two ESTs (TTL00012823 and EC269404) were retrieved from TBestDB and GenBank, respectively. The erroneous sites in the "TIGR_CYP5005A7P" sequence were indicated by red squares. The different sites between the CYP5005A7 gene and the pseudogene CYP5005A5P sequences were indicated by green squares. [file 1471-2164-10-208-S2.tiff]

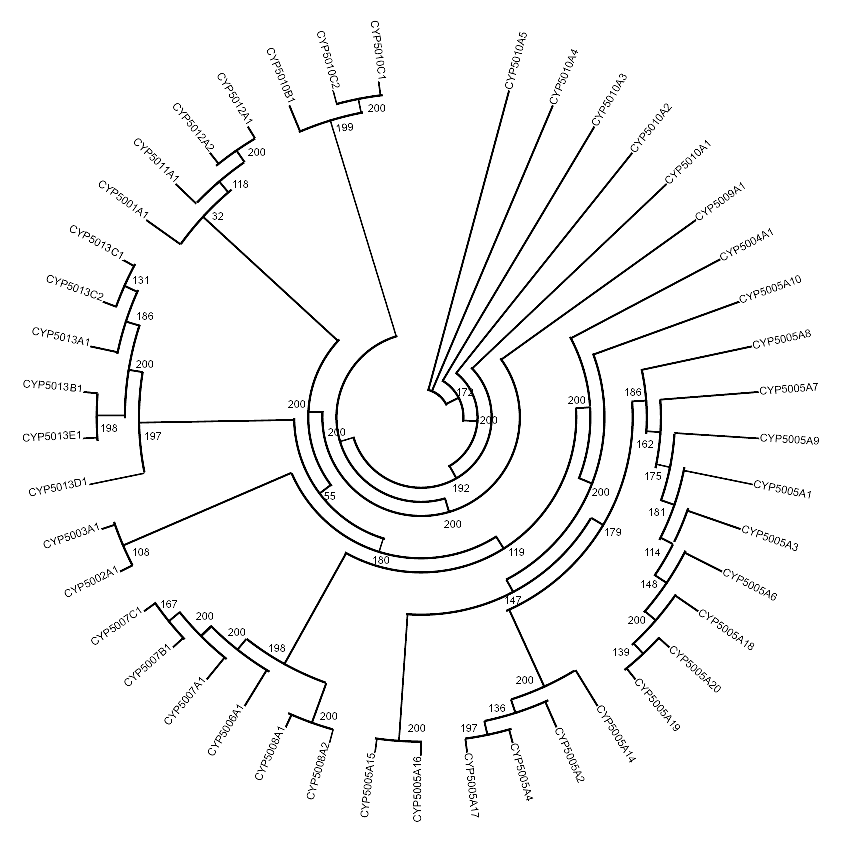

Supplement: Additional file 3 — The unrooted maximum-likelihood (ML) tree of the T. thermophila P450 protein sequences. The resulting tree was tested with 200 bootstrap repeats with PhyML and the bootstrap values are indicated on each node. [file 1471-2164-10-208-S3.tiff]

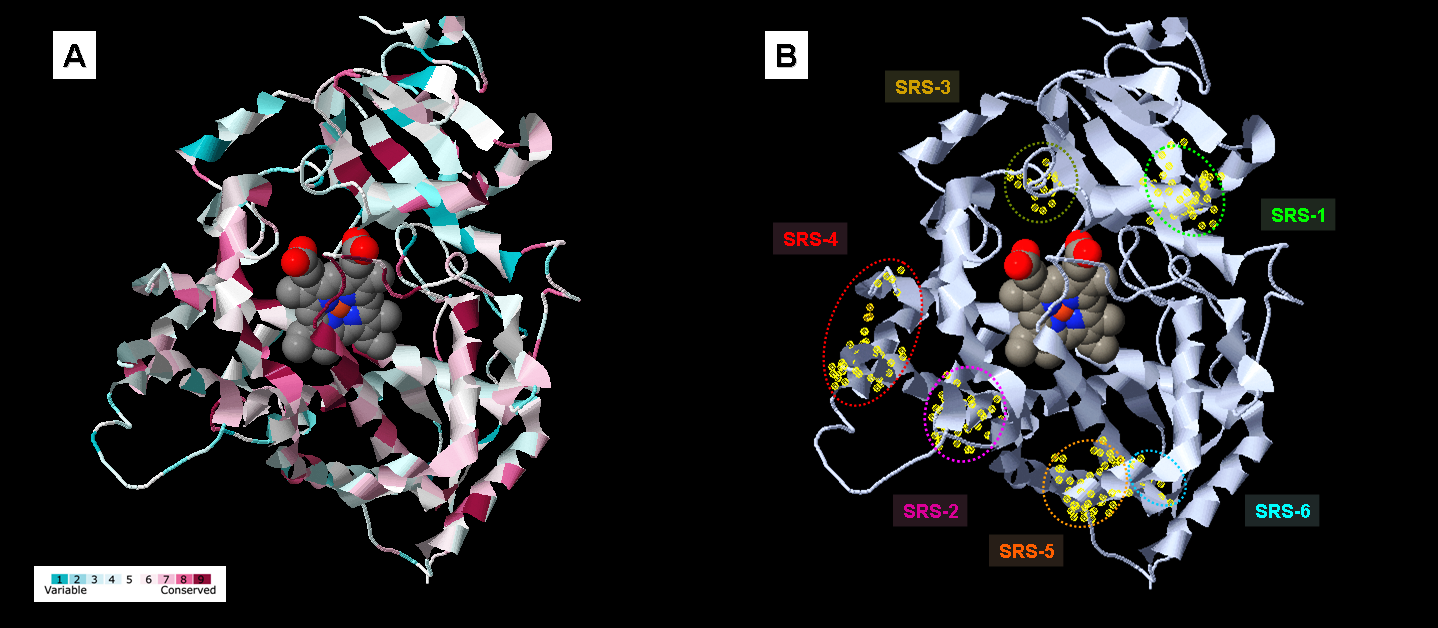

Supplement: Additional file 4 — The conservation pattern of the T. thermophila P450 family. A: The full indication of the conservation pattern inferred by Consurf based on the multiple sequence alignment using mammalian P450 CYP3A4 as the template of secondary structure elements assignment. Bottom: key to the Consurf colours; B: The six putative substrate recognition sites (SRSs) region were indicated. [file 1471-2164-10-208-S4.tiff]

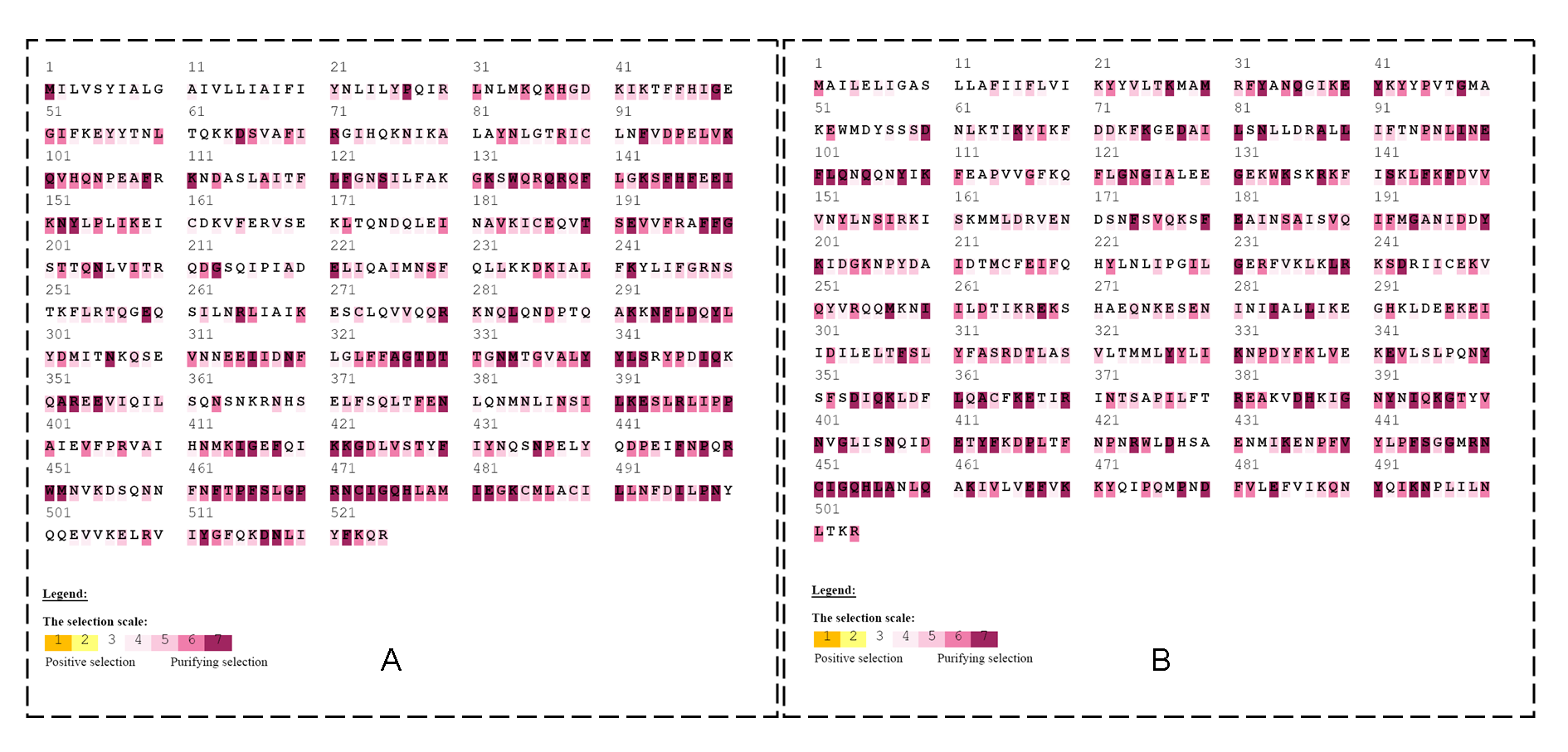

Supplement: Additional file 5 — Site-specific selection results of the CYP5005 and CYP5010 gene families. A: CYP5005 Family; B: CYP5010 Family. A seven-color scale was used by the Selecton program to represent different types of selection. Shades of yellow (colors 1 and 2) indicate ω > 1. Shades of white through magenta (colors 3 through 7) indicate various level of ω ≤ 1. [file 1471-2164-10-208-S5.tiff]

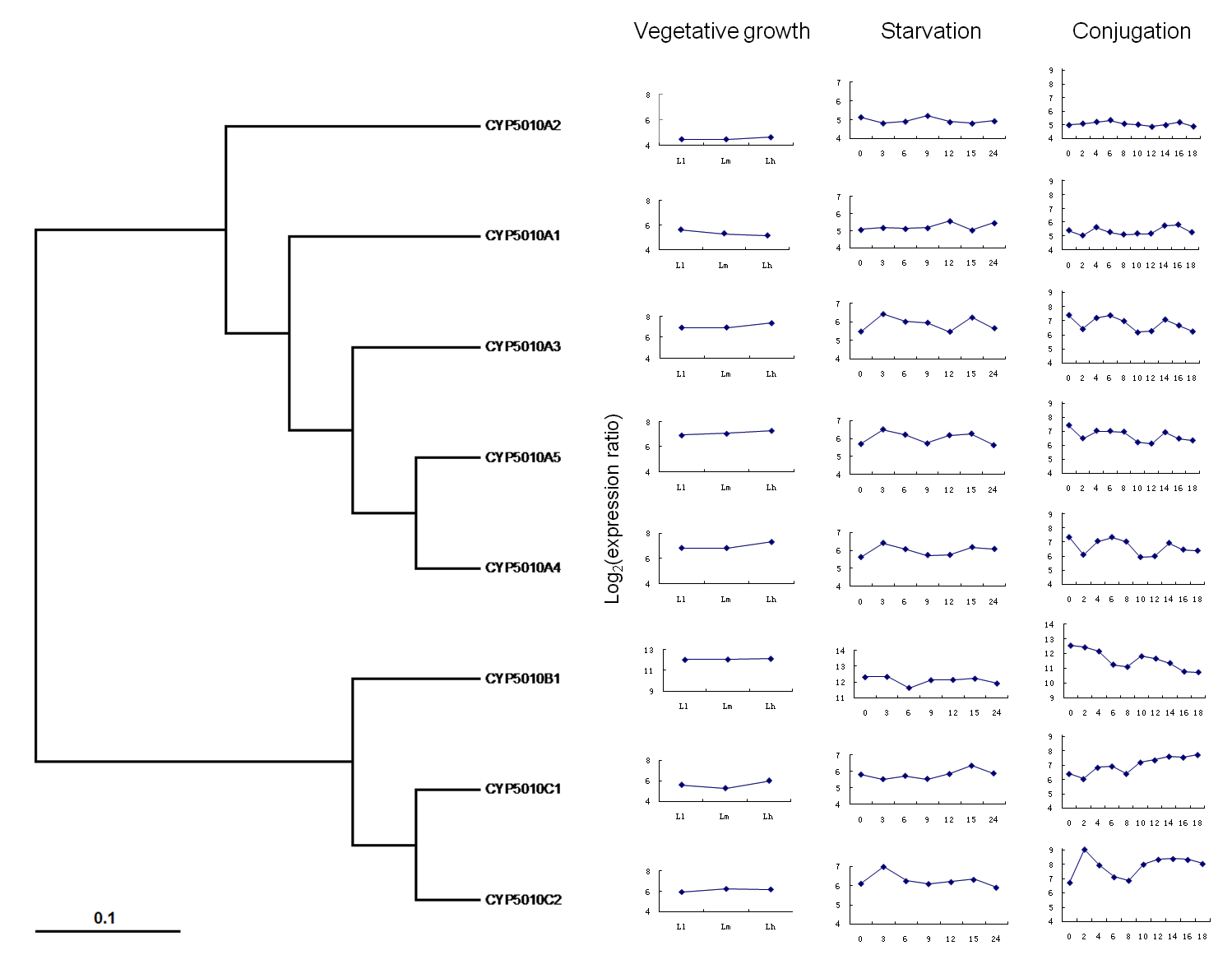

Supplement: Additional file 7 — Phylogenetic tree of the CYP5010 gene family and its expression profiles for the three physiological/developmental stages of the T. thermophila cells. Left: The ML tree of CYP5010 family used in the gene-expression evolution analysis. Relative branch lengths are proportional to number of substitutions per site. Right: The corresponding log2 transformed microarray data during three cellular conditions as a set of continuous data represent the character data at the taxa tips. [file 1471-2164-10-208-S7.tiff]
