# Supplementary material for: Genome-wide identification and characterization of cytochrome P450 monooxygenase genes in the ciliate Tetrahymena thermophila
Source: BMC Genomics. 2009 May 1;10:208. doi: 10.1186/1471-2164-10-208 (PMC2691746; doi:10.1186/1471-2164-10-208)
Supplement: Additional file 6 — Calculated values of the CYP5005 and CYP5010 gene families for each of nine different models. Calculated Akaike Information Criterion (AIC) and maximum-likelihood (ML) values of the CYP5005 and CYP5010 gene families for each of nine different models were listed. The results of best supported models under each cell conditions were marked in bold. [file 1471-2164-10-208-S6.pdf]

Additional file 6. Calculated values of the CYP5005 and CYP5010 gene families for each of nine different models

CYP5005 Family:

Cell condition	Results	PHY Dis	PHY Equ	PHY Fre	NP Dis	NP Equ	NP Fre	PUN Dis	PUN Equ	PUN Fre
Vegetative growth	AIC*	142.250	147.681	131.832	130.963	129.685	**102.917**	157.144	146.743	185.144
	ML**	-70.125	-72.840	-35.916	-64.481	-63.842	**-35.459**	-84.538	-78.871	-84.538
Starvation	AIC	353.769	374.228	359.001	326.170	330.115	**284.051**	380.115	367.562	408.115
	ML	-175.884	-186.114	-149.501	-162.085	-164.057	**-126.025**	-204.965	-198.160	-204.965
Conjugation	AIC	430.895	467.722	379.229	421.767	430.943	**352.385**	478.439	461.960	506.439
	ML	-214.448	-232.861	-159.614	-209.884	-214.472	**-160.192**	-260.647	-251.632	-260.647

CYP5010 Family:

Cell conditions	Results	PHY Dis	PHY Equ	PHY Fre	NP Dis	NP Equ	NP Fre	PUN Dis	PUN Equ	PUN Fre
Vegetative growth	AIC	32.929	67.769	54.694	**28.008**	64.401	91.418	40.895	54.458	52.895
	ML	-15.465	-32.885	-13.347	**-13.004**	-31.200	-37.709	-20.134	-27.156	-20.134
Starvation	AIC	71.561	150.533	71.266	**66.983**	147.263	224.542	81.588	121.860	93.588
	ML	-34.781	-74.267	-21.633	**-32.491**	-72.632	-104.271	-40.996	-61.741	-40.996
Conjugation	AIC	79.300	190.324	110.767	**75.138**	194.407	273.560	102.832	148.502	114.832
	ML	-38.650	-94.162	-41.384	**-36.569**	-96.204	-128.780	-52.309	-76.001	-52.309

*AIC: Akaike Information Criterion

** ML: maximum-likelihood

Models are: 1. PHY Dis = pure phylogenetic/genetic distance; 2. PHY Equ = pure hylogenetic/equal; 3. PHY Fre = pure phylogenetic/free; 4. NP Dis = nonphylogenetic/genetic distance; 5. NP Equ = nonphylogenetic/equal; 6. NP = Fre nonphylogenetic/free; 7. PUN Dis = punctuated/genetic distance; 8. PUN Equ = punctuated/equal; 9. PUN Fre = punctuated/free.
