# Supplementary material for: Genome-wide identification and characterization of cytochrome P450 monooxygenase genes in the ciliate Tetrahymena thermophila
Source: BMC Genomics. 2009 May 1;10:208. doi: 10.1186/1471-2164-10-208 (PMC2691746; doi:10.1186/1471-2164-10-208)
Supplement: Additional file 8 — Oligonucleotide primers used in this study. [file 1471-2164-10-208-S8.pdf]

Additional file 8 Oligonucleotide primers used in this study

Gene ID	Forward primer	Reverse primer
CYP5002A1	AAGTGTAGACCCAGACATGAAC	TGTTGGAAAGACGGAGTTG
CYP5005A7	GCTATCGCTTTTTGCTTTGG	GCTACTCTTGTCATGTTCCCT
CYP5005A10	AAACCTGATGGGAAAGAGA	GCTGGAGGAATCAACCTTA
CYP5005A18	CGAGGAAGGATGGAACA	TTACTTCTGTATTAGGGTTTGG
CYP5005A19	AGCAGATGGACCGATTG	TAGATGGCCCCATTATTC
CYP5006A1	GTTCCTTCTAACGGTGAGTATGT	GGAATAAAGTCGTATGGGTCT
CYP5007C1	GTTCTTCCCTTTACTTGGC	AATCCTTGCTCTGGCTTT
CYP5008A1	TCTGAAGTCGTCATCCAAACAT	GTTTTATCCCTTACTCAGCAGGTCC
CYP5010B1	CCTTCTACGGATGGTTCTG	CGGCGATCTTACTTGTTG
CYP5011A1	ATCAGGTTTATGGGGTATTT	CACCGTGTGACCAGTAGTTT
CYP5013A1	ATCCCTTATTTCAAGTTCCT	AATTGGATCTAATGGCTCTG
CYP5013D1	GGTTCTTTCTACCCTTTCG	GTTTATTCACCACGCTTTT
RT5005A6	AAGAATTGCTTGCTAGGACA	CTTCGCCACCAAAACTGA
RT5005A9	CCCCTGCCATTGATGTT	AAGTGTTAGCCAATACAGTTTCT
RT5005A8	TTGATACCACCTGCTCCC	TCCGTAGATTGTTCCCATTA
RT5005A14	GATTGGTGAATTTTCGGTT	ATAGGCTCTTGACTAGGATTAG
RT5005A2	TGAGACTTCTTCCACCTGC	TCATCGCCAAATGTTATCC
RT5005A4	TATTCCTCCTGCTATTGGTG	CCTATGTTTTATGATTTGTTTGG
RT5005A15	TACCACTGGAAATATGACAGG	TTCTAGGACCTAGTGAGAAAGG
RT5007B1	ATGATGCTTTCTTTGGCTAC	GCAGATGTCACAGATTTCG
RT5007A1	CTTCCCCAAGAGTCACC	GAGGGCTAAATGCTGTCC
RT5010A1	TTTCTTGGAAACGGTATTG	AATCTATGGCATCGTATGAGT
RT5010A2	AAATACAAGCGCCCCAA	GCAGTTTCTCATTCCACCAG
5013A1Int	TATATGTTGTCAATCTTGGTAATAAAA	GCTTTATCTTAGAGCCTATTTTTTAAG
5005A7_Genome	ACCTGGAACATCTATCTAACTCA	AAACAACATAATGGAATCCTTAA
