# Supplementary material for: Genome-wide identification and characterization of cytochrome P450 monooxygenase genes in the ciliate Tetrahymena thermophila
Source: BMC Genomics. 2009 May 1;10:208. doi: 10.1186/1471-2164-10-208 (PMC2691746; doi:10.1186/1471-2164-10-208)
Supplement: Additional file 9 — Codon usage parameters of 44 T. thermophila P450 genes. The CAI, ENc, GC content, GC12, GC3s and the RSCU values of the two most important axes were listed. [file 1471-2164-10-208-S9.pdf]

Additional file 9. Codon usage parameters of *T. thermophila* P450 genes

Gene ID	CAI	ENc	GC	GC1	GC2	GC12	GC3s	Axis1	Axis2
CYP5001A1	0.391	42.56	0.292	0.393	0.251	0.322	0.232	-0.13417	-0.10572
CYP5002A1	0.376	47.00	0.295	0.373	0.272	0.3225	0.240	-0.05981	0.1136
CYP5003A1	0.410	39.08	0.267	0.367	0.234	0.3005	0.200	-0.07981	0.19395
CYP5004A1	0.403	42.57	0.293	0.400	0.245	0.3225	0.234	-0.04556	-0.27053
CYP5005A1	0.393	43.18	0.286	0.374	0.267	0.3205	0.217	-0.08977	0.00701
CYP5005A2	0.378	41.64	0.280	0.355	0.266	0.3105	0.219	-0.12121	-0.18034
CYP5005A3	0.408	39.79	0.292	0.402	0.257	0.3295	0.217	-0.03047	0.05393
CYP5005A4	0.381	43.06	0.277	0.369	0.259	0.314	0.203	-0.10783	-0.05525
CYP5005A6	0.384	41.55	0.286	0.379	0.274	0.3265	0.205	-0.13596	-0.07426
CYP5005A7	0.397	42.04	0.276	0.345	0.260	0.3025	0.223	0.00211	0.01118
CYP5005A8	0.419	41.91	0.301	0.378	0.255	0.3165	0.270	-0.00587	-0.02631
CYP5005A9	0.371	45.82	0.282	0.368	0.260	0.314	0.218	-0.0254	0.03544
CYP5005A10	0.397	42.31	0.283	0.358	0.251	0.3045	0.240	-0.06007	0.13972
CYP5005A14	0.414	42.18	0.307	0.391	0.292	0.3415	0.238	-0.0214	-0.09128
CYP5005A15	0.385	41.98	0.285	0.374	0.272	0.323	0.209	-0.10133	0.07658
CYP5005A16	0.392	40.30	0.264	0.359	0.262	0.3105	0.171	-0.12858	0.11503
CYP5005A17	0.384	43.09	0.299	0.401	0.266	0.3335	0.230	-0.05489	0.14244
CYP5005A18	0.371	42.05	0.291	0.378	0.287	0.3325	0.208	-0.11761	0.06623
CYP5005A19	0.378	43.67	0.277	0.357	0.273	0.315	0.201	-0.04788	-0.04715
CYP5005A20	0.393	45.79	0.298	0.382	0.288	0.335	0.224	0.03403	0.18147
CYP5006A1	0.446	42.25	0.287	0.356	0.263	0.3095	0.242	0.05212	0.03689
CYP5007A1	0.469	44.13	0.302	0.371	0.246	0.3085	0.289	0.16979	-0.00481
CYP5007B1	0.450	45.23	0.29	0.348	0.261	0.3045	0.261	0.06916	0.0531
CYP5007C1	0.419	40.42	0.275	0.342	0.250	0.296	0.233	0.00138	-0.02996
CYP5008A1	0.666	41.99	0.372	0.409	0.278	0.3435	0.429	0.85988	0.01997
CYP5008A2	0.401	43.80	0.299	0.381	0.252	0.3165	0.264	0.01763	0.04078
CYP5009A1	0.395	41.59	0.285	0.378	0.248	0.313	0.229	-0.0627	0.07809
CYP5010A1	0.378	42.91	0.267	0.361	0.229	0.295	0.211	-0.1105	0.06684
CYP5010A2	0.417	40.07	0.269	0.371	0.237	0.304	0.199	-0.12301	-0.07483
CYP5010A3	0.407	41.55	0.279	0.386	0.247	0.3165	0.204	-0.11424	-0.27547
CYP5010A4	0.412	40.83	0.276	0.383	0.247	0.315	0.198	-0.10933	-0.25547
CYP5010A5	0.408	41.47	0.278	0.383	0.247	0.315	0.204	-0.11317	-0.27225
CYP5010B1	0.654	40.64	0.333	0.390	0.264	0.327	0.345	0.4895	-0.05112
CYP5010C1	0.441	43.44	0.302	0.403	0.265	0.334	0.238	0.0433	0.01466
CYP5010C2	0.467	44.96	0.302	0.413	0.261	0.337	0.232	0.14909	-0.15695
CYP5011A1	0.396	41.33	0.274	0.345	0.273	0.309	0.204	-0.03955	0.16726
CYP5012A1	0.402	42.03	0.277	0.362	0.253	0.3075	0.216	-0.14423	0.21241
CYP5012A2	0.403	39.33	0.276	0.370	0.263	0.3165	0.195	-0.07433	-0.01078
CYP5013A1	0.422	42.24	0.313	0.418	0.292	0.355	0.229	0.06643	0.1044
CYP5013C1	0.386	41.06	0.272	0.34	0.275	0.3075	0.201	-0.14295	0.0756
CYP5013B1	0.396	40.79	0.271	0.349	0.230	0.2895	0.234	-0.10456	-0.00144
CYP5013C2	0.428	37.62	0.277	0.371	0.278	0.3245	0.182	-0.12791	-0.05733
CYP5013D1	0.618	41.17	0.365	0.450	0.285	0.3675	0.360	0.75604	-0.03311
CYP5013E1	0.415	41.15	0.271	0.361	0.255	0.308	0.197	-0.09824	0.0696
